# Oligonucleotide Tagging for Copper-Free Click Conjugation

**DOI:** 10.3390/molecules18077346

**Published:** 2013-06-24

**Authors:** Anup M. Jawalekar, Sudip Malik, Jorge M. M. Verkade, Brian Gibson, Nancy S. Barta, John C. Hodges, Alan Rowan, Floris L. van Delft

**Affiliations:** 1 Radboud University Nijmegen, Institute for Molecules and Materials, Heyendaalseweg 135, 6525 AJ Nijmegen, The Netherlands; E-Mails: jawanup@yahoo.com (A.M.J.); psusm2@iacs.res.in (S.M.); a.rowan@science.ru.nl (A.R.); 2 SynAffix BV, Heyendaalseweg 135, 6525 AJ Nijmegen, The Netherlands; E-Mail: j.verkade@synaffix.com; 3 Berry and Associates, Inc., 2434 Bishop Circle East, Dexter, MI 48130, USA; E-Mails: bgibson@berryassoc.com (B.G.); nbarta@berryassoc.com (N.S.B.); jhodges@berryassoc.com (J.C.H.)

**Keywords:** copper-free click, oligonucleotide conjugation, bicyclononyne, azide, strain-promoted cycloaddition

## Abstract

Copper-free click chemistry between cyclooctynes and azide is a mild, fast and selective technology for conjugation of oligonucleotides. However, technology for site-specific introduction of the requisite probes by automated protocols is scarce, while the reported cyclooctynes are large and hydrophobic. In this work, it is demonstrated that the introduction of bicyclo[6.1.0]nonyne (BCN) into synthetic oligonucleotides is feasible by standard solid-phase phosphoramidite chemistry. A range of phosphoramidite building blocks is presented for incoporation of BCN or azide, either on-support or in solution. The usefulness of the approach is demonstrated by the straightforward and high-yielding conjugation of the resulting oligonucleotides, including biotinylation, fluorescent labeling, dimerization and attachment to polymer.

## 1. Introduction

Synthetic DNA and RNA oligonucleotides (ONs) are key tools in a broad variety of diagnostic and therapeutic applications, including microarray technology [1], antisense and gene-silencing therapies [2], nanotechnology [3] and materials sciences [4,5]. Generally, such applications require the introduction of a suitable handle in an oligonucleotide to enable selective conjugation to a functionality of interest [6,7,8]. For example, attachment of a cell-penetrating ligand is the most commonly applied strategy to tackle the low internalization rate of ONs into target cells [2], currently the main bottleneck in oligonucleotide-based therapeutics (antisense, siRNA). Similarly, the preparation of oligonucleotide-based microarrays requires the selective immobilization of ONs on a suitable solid surface, e.g., glass [1]. Conventional post-synthetic labeling protocols, based on amide bond formation or sulfide-based chemistry [8,9,10] typically suffer from low yield and long reaction times and often require a high concentration of the biomolecule in combination with a large excess of coupling partner. One promising alternative to the traditional conjugation technologies involves the copper-catalyzed cycloaddition of alkynes and azides, a procedure commonly referred to as “click reaction” [11,12]. However, the use of copper for oligonucleotide conjugation may be compromised due to potential metal-catalyzed strand degradation and/or difficulties in final purification [13,14,15]. Although new ligands reduce the chance of undesired chain cleavage during copper-catalyzed click reaction [16,17,18], strain-promoted azide-alkyne cycloaddition (SPAAC) offers the possibility of oligonucleotide conjugation in the absence of copper [19,20,21,22,23] as demonstrated for oligonucleotides labeled with plain cyclooctyne (OCT) [15] or the more reactive dibenzofused cyclooctyne DIBO [24]. Most recently, Brown *et al*. [25,26] further extended the latter approach by ON incorporation of aminoalkyl thymidine derivatives, followed by selective *N*-acylation with azide or cyclooctyne after cleavage from support. Alternative approaches for the preparation of azide-containing nucleotides—compromised by the incompatibility of azide with phosphoramidite chemistry—involve post-synthetic nucleophilic substitution [27,28,29] or selective diazotransfer reaction [30] or phosphonate-based coupling chemistry [31,32,33,34] However, a simple and general strategy for the on-support, automated synthesis of oligonucleotides with readily accessible building blocks, and suitable for introduction of any functional group (including cyclooctyne and azide), is still desirable.

**Figure 1:**
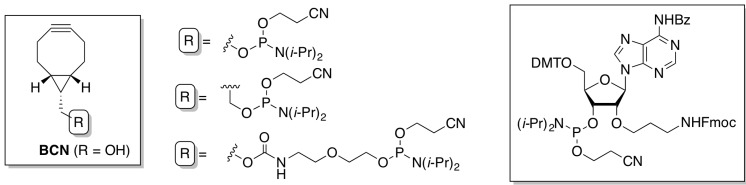
Structures of BCN- and adenosine-based phosphoramidites for incorporation into and copper-free conjugation of oligonucleotides.

We here report two versatile approaches for conjugation of oligonucleotides by strain-promoted azide-alkyne cycloaddition. First, a range of novel phosphoramidite building blocks was developed for incorporation of bicyclo[6.1.0]nonyne (BCN) [35] and an adenosine-based building block is presented suitable for BCN or azide introduction following standard oligonucleotide synthesis protocols, and allowing *multiple* nucleotide 2′-functionalization (Figure 1). The ease of operation of copper-free click conjugation is demonstrated for a range of functional groups, by oligonucleotide dimerization, and by the preparation and characterization of an amphiphilic polythiophene-oligonucleotide hybrid polymer.

## 2. Results and Discussion

### 2.1. 5′-Labeling and Conjugation of Oligonucleotides

#### 2.1.1. Preparation of BCN-Phosphoramidites

**Scheme 1:**
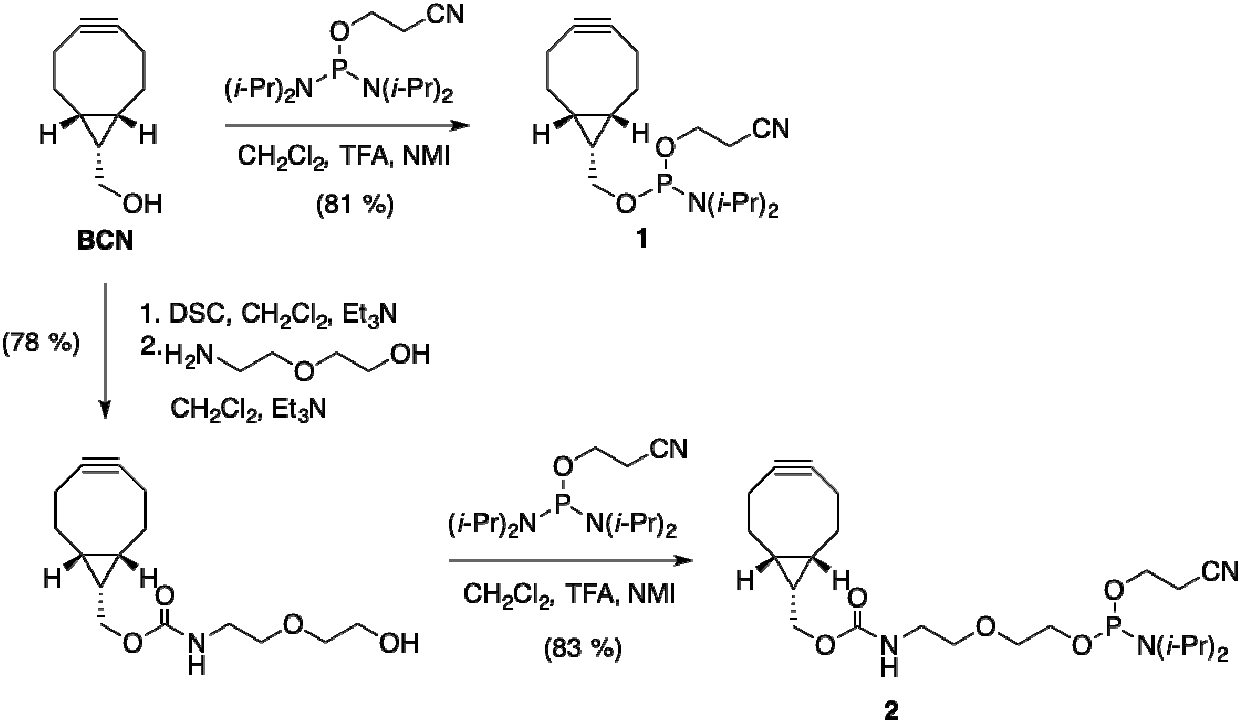
Synthesis of BCN-phosphoramidites **1** and **2**.

Earlier reported approaches for copper-free conjugation of oligonucleotides were based solely on DIBO, a dibenzofused cyclooctyne that inevitably leads to a mixture of regioisomeric and diastereomeric adducts upon reaction with azide. We reasoned that bicyclo[6.1.0]nonyne (BCN) has particular potential for oligonucleotide conjugation, due to its higher reactivity in comparison to DIBO, its relatively low lipophilicity in comparison all dibenzofused cyclooctynes [36], and its plane-symmetry, which precludes the formation of regioisomers upon cycloaddition. Thus, phosphoramidite derivative **1** was, prepared in a single step from commercially available BCN alcohol (R=OH) with 81% yield (Scheme 1), as well as a diethyleneglycol chain-extended phosphoramidite **2**. 

#### 2.1.2. Activation and Incorporation of BCN-Phosphoramidites

**Scheme 2:**
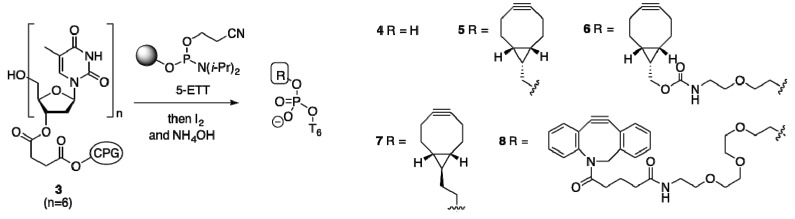
Solid-phase synthesis of BCN-charged oligonucleotides **5**–**8**.

**Scheme 3:**
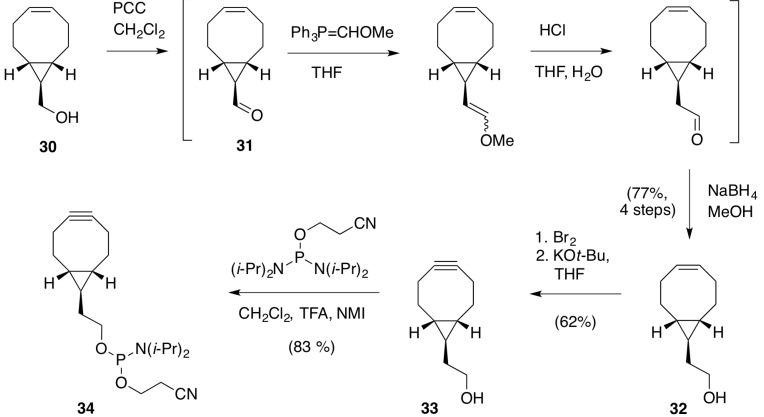
Synthesis of *exo*-BCN-ethanol phosphoramidite for the preparation of **7**.

Next, compound **1** was activated with 5-ethylthiotetrazole (ETT) and attached to hexathymidine nucleotide (**3**, n = 6), supported on controlled pore glass (Scheme 2), leading to a single ON after oxidation and cleavage from support, as indicated by HPLC. However, mass spectrometry indicated that not the expected BCN-containing diester **5**, but 5′-monophosphate **4** had been isolated instead. We attribute the formation of **4** to rapid hydrolysis of the transiently formed diester phosphate **5**, presumably involving a heterolytic cleavage mechanism with formation of a surprisingly stable BCN-derived cyclopropylmethyl cation [37]. Based on the latter assumption, phosphoramidite **2** was next subjected to the same oligonucleotide synthesis protocol, now leading to the isolation of the desired 5′-BCN-containing hexanucleotide **6** in high yield. Another successful strategy to avoid the formation of a cyclopropylmethyl cation involved the preparation of hexathymidine conjugate ON **7**, containing a homologated BCN ethyl derivative (Scheme 3).

#### 2.1.3. Comparison of BCN-Containing Oligonucleotides to Dibenzofused Cyclooctynes

**Figure 2:**
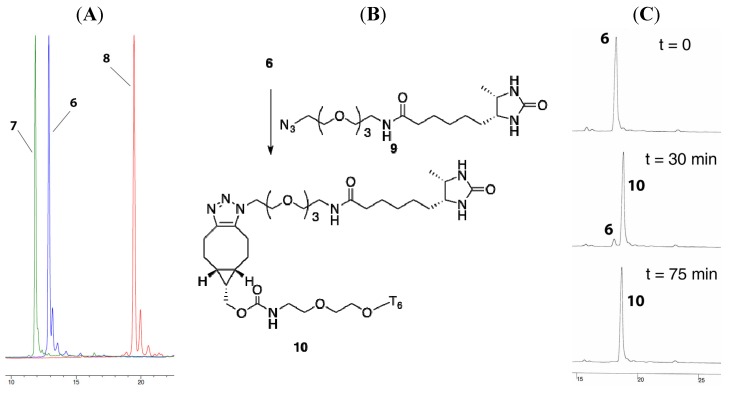
(**A**) HPLC-reversed phase profile of **6**–**8**. (**B**) Formation of **10** by SPAAC dimerization of **6** and **9**. (**C**) HPLC-traces of conversion of **6** into **10** upon the addition of **9**.

Now the stage was set to compare the lipophilicity of the oligonucleotides **6** and **7** containing a BCN-type cyclooctyne to a dibenzoannulated cyclooctyne. To this end, we prepared DBCO-containing hexa-T (compound **8** in Scheme 2) from commercially available DBCO-phosphoramidite. To our satisfaction, C18-reversed phase HPLC analysis confirmed the higher polarity of BCN-containing ONs **6** and **7** (elution after 13.2 and 12.0 min, respectively) with respect to DBCO-containing ON **8** (19.6 min). The usefulness of BCN-containing ONs for metal-free cycloaddition with azide was also evaluated, by addition of desthiobiotin azide **9** to BCN-charged ON **7** (Figure 2B). HPLC analysis indicated a rapid and quantitative cycloaddition of **6** and **9** (Figure 2C, Figure S1), to give the expected triazole adduct **10** in only 75 min, thereby corroborating the usefulness of 5′-BCN incorporation for copper-free conjugation of oligonucleotides.

### 2.2. 3′-Fmoc-Aminopropyl Adenosine for Internal Labeling and Conjugation of Oligonucleotides

Despite the promising results with BCN-derived phosphoramidites for the preparation of 5′-functionalized ONs, the introduction of BCN at other positions in an oligonucleotide (3′-end or internally) is not readily accessible with simple building blocks. Moreover, it is clear that a phosphoramidite-based strategy for the introduction of an azide group, the complementary partner for SPAAC, is hampered by competitive Staudinger reduction of azide with P^III^-type reagents [27,31]. Therefore, our next aim was to develop a generic building block for internal incorporation in an ON chain, to facilitate subsequent on-support derivatization with any functional group of choice, including BCN or azide.

#### 2.2.1. Preparation, Incorporation and Model Studies

**Scheme 4:**
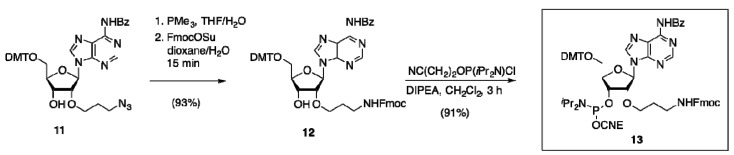
Synthesis of Fmoc-aminopropyl adenosine **13**.

Based on the above reasoning, a straightforward synthetic route towards Fmoc-protected 2′-O-aminopropyl adenosine-based building block **13** was designed (Scheme 4). Importantly, the 2′-aminopropyl group would ensure subsequent selective functionalization after Fmoc-deprotection. A similar strategy was recently reported based on a 2′-aminoethyl thymidine building block [25], but to the best of our knowledge on-support oligonucleotide functionalization has not been reported to date. An advantage of modification via 2′-OH of ribose, instead of conjugation via the nucleobase, is that negligible interference with hybridization is expected, due to the direction of a 2′-O-functional group towards the minor groove of a DNA duplex. 

Thus, starting phosphoramidite **13** was conveniently prepared in only three high-yielding steps from readily available 2′-(3-azidopropyl) adenosine building block **11** [38] by Staudinger reduction, Fmoc-protection and phosphitylation (Scheme 4). 

**Scheme 5:**
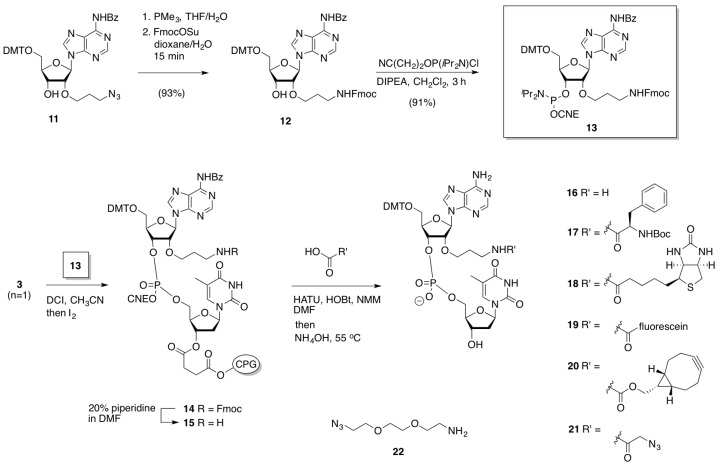
On-support incorporation and conjugation of **13** into a dinucleotide.

Next, CPG-immobilized thymidine (**3**) was 5′-chain extended with building block **13** (Scheme 5) under standard conditions. The successful formation of the projected phosphate diester **14** was corroborated by Fmoc removal (20% piperidine in DMF) to give intermediate **15**, followed by cleavage from CPG, leading to the free amino-derivative **16** in high purity, as confirmed by HPLC and HRMS (Figure S2 and Table S1). Alternatively, the Fmoc-deprotected dinucleotide **15** was subjected to on-support acylation before cleavage with NH_4_OH, thereby generating a range of DMT-on dinucleotides functionalized with phenylalanine (**17**), biotin (**18**) or fluorescein (**19**). Similarly, intermediate **15** could be smoothly and cleanly converted into BCN-charged carbamate derivative **20**, or azide-containing dinucleotide **21**.

The usefulness of the BCN-charged oligonucleotide for follow-up functionalization by SPAAC was in this case corroborated by two experiments. First, treatment of **20** with excess of 2-(2-(2-azidoethoxy)ethoxy)ethanamine (**22**) afforded the anticipated triazole adduct in quantitative yield (Figure S2 and Table S1). A similar smooth reaction was observed for dimerization of BCN-charged dinucleotide **20** and azide-charged dinucleotide **21**, affording exclusively the (3+2) cycloaddition product, as confirmed by HPLC and LC-MS analysis (Figure S2 and Table S1). 

#### 2.2.2. Oligonucleotide Dimerization

**Scheme 6:**
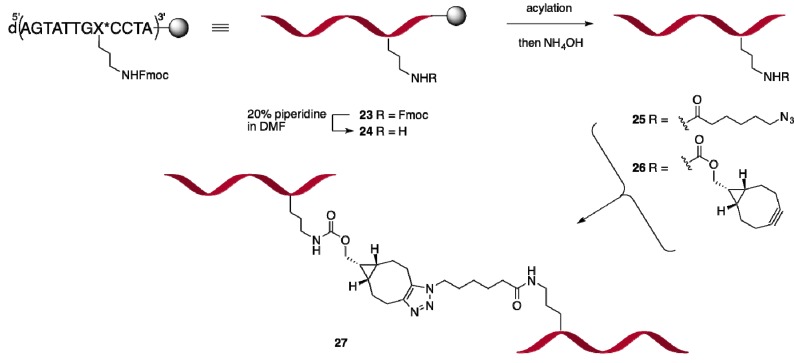
On-support preparation of azide or BCN-charged oligonucleotides and dimerization in solution.

Now the stage was set to evaluate the scope of building block **13** for the synthesis and functionalization of longer oligonucleotides, and to explore the usefullness of SPAAC to obtain site-specifically conjugated oligonucleotides (Scheme 6). As anticipated, attachment of Fmoc building block **13** to a CPG-tetranucleotide, and subsequent repetitive coupling with standard ON building blocks, proceeded smoothly to give the 12-mer ON **23** with sequence d(AGTATTGX*CCTA) (X* = 2′-Fmoc-*N*-propyladenosine), as corroborated by cleavage from CPG of an analytical sample. Next, undecanucleotide **23** was on-support Fmoc-deprotected with piperidine (to **24**) and coupled with azidohexanoic acid or a BCN-derived active carbonate and cleaved from support, to give the respective azide derivative **25** and BCN-derivative **26**, respectively. As anticipated, overnight stirring of a 1:1 mixture of the BCN- and the azide-functionalized ON conjugates **26** and **25**, respectively, was found to give the desired oligonucleotide dimer **27**, as confirmed by HPLC and MALDI-TOF analysis (Table S2), thereby demonstrating for the versatility of our approach for conjugation of oligonucleotides at any adenosine in the ribose backbone.

#### 2.2.3. Oligonucleotide-Polythiophene Hybrid

**Figure 3:**
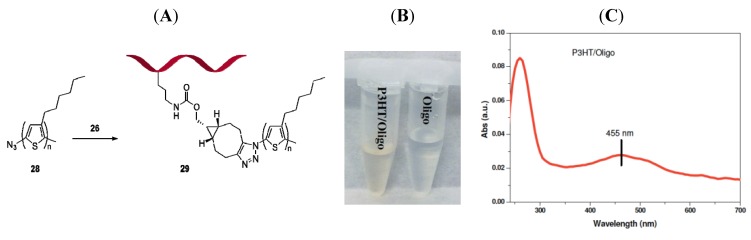
(**A**) Conjugation of azido-terminated P3HT **28** and BCN-containing oligonucleotide **26** leading to **29**. (**B**) Aqueous solution of **28** turns yellow as a consequence of spontaneous (3+2) cycloaddition leading to solubilization. (**C**) UV-VIS spectrum of aqueous solution shows the appearance of the typical absorption band of P3HT at 455 nm.

Finally, we were intrigued by the idea of applying SPAAC for the synthesis of functional oligonucleotide-containing materials, in particular toward the construction of bioresponsive films. Poly(3-hexylthiophene) (P3HT) is a well known electroconductive material which can be found in solar cells and other nanoelectronic devices but is known to be notoriously insoluble in aqueous systems. One potential strategy for solubilization would involve attachment of oligonucleotides to these synthetic lipophilic polymers. To this end, azido-functionalized P3HT (**28**) was treated with BCN-conjugated ON 5′-d(AGTATTGXCCTA)-3′ (**26**) and the reaction was monitored by color change as well as UV-VIS spectroscopy (Figure 3). To our satisfaction, mixing **28** and **26** led to the formation of a yellowish solution, thereby indicating the formation of composites through (3+2) cycloaddition between P3HT and ON as the result of the slow solubilization of the otherwise completely water-insoluble hydrophobic polymer **28**. More conclusive support for succesfull conjugation was obtained by UV-VIS spectroscopy, which clearly revealed the presence of P3HT in the aqueous solution as indicated by the appearance of absorption peak at 455 nm (Figure 3C), as well as by MALDI-TOF analysis (Figure S4).

## 3. Experimental

^1^H-NMR spectra were recorded on Bruker DMX 300 or Varian Inova-400 spectrometers at 300 K. TMS (*δ*_H_ 0.00) or CD_3_CN (*δ*_H_ 1.94) was used as the internal reference. ^13^C-NMR spectra were recorded in CDCl_3_ at 75 MHz on a Bruker DMX 300 spectrometer, using the central resonance of CDCl_3_ (*δ*_C_ 77.0) as the internal reference. ^31^P NMR and ^1^H-NMR spectra for **1**, **2** and **V** were provided by NuMega Resonance Labs. Mass spectra were obtained on Applied Biosystems Voyager DE-Pro MALDI-TOF (no calibration) or JEOL AccuToF. Identities of **4**, **5**, **6**, **7**, **8**, and **10** were confirmed by MS or LCMS that were provided by Novatia, LLC. Except as specified below, chemicals were purchased from Aldrich Chemical Co. and used without further purification. CH_2_Cl_2_, acetonitrile, THF, Et_2_O and toluene were obtained dry from a MBRAUN SPS-800 solvent purification system; and CH_3_OH was distilled from magnesium and iodine. Aqueous solutions are saturated unless otherwise specified. All reactions were performed under anhydrous conditions under argon and monitored by TLC on Kieselgel 60 F254 (Merck, Darmstadt, Germany). Prior to analysis of phosphoramidites, TLC plates were buffered by dipping in 2% Et_3_N in hexanes and air dried. Detection was by examination under UV light (254 nm) and by charring with 10% sulfuric acid in methanol, with aqueous KMnO_4_, or ethanolic phosphomolybdic acid (PMA). Silica gel (Acros 0.035–0.070 mm, and for phosphoramidites, SiliCycle, 0.060–0.200 mm) was used for chromatography. (*i-*Pr_2_N)_2_POCH_2_CH_2_CN was obtained from Digital Specialty Chemicals, catalog # 269 (2-Cyanoethyl *N,N,N′N′*-tetraisopropylphosphorodiamidite). Compound **9** was provided by Berry and Associates, Inc., catalog # BT 1075 (Desthiobiotin-TEG azide). DBCO-phosphoramidite was obtained from Glen Research Corp., catalog #10-1941 (5′-DBCO-TEG phosphoramidite). 

Automated ON synthesis was completed using a Millipore Expedite (8900 series) nucleic acid synthesis system using the recommended thymidine conditions for each synthesis cycle in a DMT-on protocol for 0.2 Mol columns. Standard synthesizer reagents and thymidine-CPG were obtained from Glen Research Corporation. The ONs were deprotected and cleaved from the CPG support by manually passing conc. NH_4_OH back and forth through the column with a pair of syringes for 15 min. The resulting ON solutions were sparged with N_2_ for 3 hours to remove excess NH_3_. The concentrated solutions were frozen and lyophilized.

*2-(endo-Bicyclo[6.1.0]non-4-yn-9-yl)methyl (2-cyanoethyl) diisopropylphosphoramidite* (**1**) *Endo*-BCN-methanol (1.00 g, 6.66 mmol) was dissolved in anhydrous CH_2_Cl_2_ (17 mL) and treated with (*i-*Pr_2_N)_2_POCH_2_CH_2_CN (2.43 mL, 7.66 mmol). A solution of CF_3_CO_2_H (0.25 M) and 1-methylimidazole (0.50M) in anhydrous CH_2_Cl_2_ (13.3 mL) was added dropwise then the reaction was stirred at RT for 90 min. TLC (hexane-acetone, 90:10, PMA stain) shows the complete consumption of endo-BCN-methanol (Rf 0.40) and the appearance of a single new product (Rf 0.68). The reaction mixture was partitioned between CH_2_Cl_2_ (50 mL) and H_2_O (50 mL). The organic layer was washed with H_2_O (50 mL) and saturated NaHCO_3_ (35 mL). The organic layer was dried over Na_2_SO_4, _filtered and concentrated at reduced pressure. Silica (36 g) was slurried in hexane-Et_3_N (95:5, 85 mL) and packed into a 60 mL sintered glass funnel. The bed of silica was eluted with hexanes (50 mL) before a solution of crude product in hexanes (7 mL) was applied. Elution with hexane-acetone (96:4), collecting 30 mL fractions affords purified **1** (1.88 g, 80.6%) after evaporation of solvent and drying under vacuum overnight. ^1^H-NMR (500 MHz, CD_3_CN) δ 3.82–3.67 (m, 4H), 3.65–3.55 (m, 2H), 2.62 (q, 2H), 2.29–2.18 (m, 6H), 2.14 (s, 2H), 1.35–1.27 (m, 1H), 1.17 (d, 12H), 0.90–0.82 (2H). ^31^P NMR (500 MHz, CD_3_CN) δ 147.64 (s, 98.6%). MS (AP+): 351 (M+H); 373 (M+Na).

*endo-Bicyclo[6.1.0]non-4-yn-9-ylmethyl (2-(2-hydroxyethoxy)ethyl)carbamate* Under an atmosphere of argon, BCN (1.80 g; 12.0 mmol) was dissolved in 100 mL anhydrous MeCN, and disuccinimidylcarbonate (3.38 g; 13.2 mmol) and triethylamine (5.0 mL; 3.64 mmol; 36.0 mmol) were added. The resulting mixture was stirred for 2.5 h and concentrated. The residue was taken up in diethyl ether (100 mL) and washed with a saturated aqueous solution of NH_4_Cl. After separation, the organic layer was dried (Na_2_SO_4_) and concentrated. The residue was taken up in DCM (100 mL), washed with water (50 mL), dried (Na_2_SO_4_) and concentrated. The residue was taken up in EtOAc (50 mL) and concentrated. Crude carbonate: 3.08 g.

The crude residue (2.5 g, max 8.6 mmol) was dissolved in DCM (100 mL) under an atmosphere of argon. 2-(2-Aminoethoxy)ethanol (1.0 mL; 1.1 g; 10.3 mmol) and triethylamine (3.6 mL: 2.6 g; 25.8 mmol) were added and the resulting mixture was stirred for 15 min. After concentration, the residue was purified by column chromatography (EtOAc/pentane 3/1). The pure fractions were combined, concentrated, taken up in EtOAc, filtered and concentrated. Yield over two steps: 1.48 g (5.26 mmol; 54%). ^1^H-NMR (CDCl_3_, 300 MHz): δ (ppm) 5.04 (bs, 1H), 4.16 (d, J = 8.1 Hz, 2H), 3.79–3.70 (m, 2H), 3.62–3.54 (m, 2H), 3.40 (dd, J = 10.7 Hz, 5.4 Hz), 2.37–2.12 (m, 6H), 2.01 (bs, 1H), 1.70–1.48 (m, 2H), 1.43–1.30 (m, 1H), 1.02–0.87 (m, 2H).

*endo-Bicyclo[6.1.0]non-4-yn-9-ylmethyl(2-(2-(((2-cyanoethoxy)(diisopropylamino)-phosphino)oxy) ethoxy)ethyl)carbamate* (**2**) *Endo-*bicyclo[6.1.0]non-4-yn-9-ylmethyl (2-(2-hydroxyethoxy)ethyl) carbamate (5.00 g, 17.8 mmol) was dissolved in anhydrous CH_2_Cl_2_ (65 mL) and treated with (*i-*Pr_2_N)_2_POCH_2_CH_2_CN (6.49 mL, 20.4 mmol). A solution of CF_3_CO_2_H (0.25 M) and 1-methylimidazole (0.50 M) in anhydrous CH_2_Cl_2_ (35.5 mL) was added dropwise then the reaction was stirred at RT for 90 min. TLC (hexane-acetone, 80:20, PMA stain) shows the complete consumption of starting material (Rf 0.15) and the appearance of a single new product (Rf 0.50). The reaction mixture was partitioned between CH_2_Cl_2_ (50 mL) and H_2_O (150 mL). The organic layer was washed with H_2_O (150 mL) and 5% NaHCO_3_ (150 mL). The organic layer was dried over Na_2_SO_4, _filtered and concentrated at reduced pressure. Silica (130 g) was slurried in hexane-acetone-Et_3_N (95:5:5, 250 mL) and packed into a 5 cm diameter column. The crude product was dissolved in CH_2_Cl_2_ (8 mL) and diluted with hexanes (12 mL) and the cloudy solution was applied to the column. Initial elution was performed with hexane-acetone-CH_2_Cl_2_-Et_3_N (79.5:10:10:0.5, 100 mL). Subsequent elution was performed with Hexane-Acetone-Et_3_N (85.5:14:0.5), collecting 75 mL fractions. Pure product fractions were combined and concentrated at reduced pressure. The resulting colorless liquid was re-dissolved in CH_2_Cl_2_ (50 mL) and concentrated again. Further drying overnight under vacuum affords purified **2** (6.86 g, 80%). ^1^H-NMR (500 MHz, CD_3_CN) δ 5.17 (s, 1H), 4.13 (d, 2H), 3.82–3.51 (m, 8H), 3.38–3.26 (m, 2H), 2.64 (q, 2H), 2.29–2.18 (m, 6H), 2.14 (s, 2H), 1.38–1.22 (m, 1H), 1.17 (d, 12H), 0.88–0.84 (2H). ^31^P NMR (500 MHz, CD_3_CN) δ 149.11 (s, 100%). MS (AP+): 482 (M+H); 504 (M+Na).

*(Z)-exo-Bicyclo[6.1.0]non-4-ene-9-carbaldehyde* (**31**) (Z)-*exo*-Bicyclo[6.1.0]non-4-en-9-ylmethanol (**30**) (5.2 g, 26.6 mmol) was dissolved in DCM (300 mL). Pyridinium chlorochromate (10.5 g, 48.5 mmol) was added. The resulting reaction mixture was stirred for 2 h and subsequently filtered over a short path of silica gel. The filtrate was concentrated and purified by column chromatography (DCM), yielding 4.95 g of the aldehyde (B). ^1^H NMR (300 MHz, CDCl_3_) δ (ppm) 9.04 (d, *J* = 5.5 Hz, 1H), 5.71–5.58 (m, 2H), 2.49–2.01 (m, 6H), 1.77–1.44 (m, 5H).

*(Z)-exo-2-(Bicyclo[6.1.0]non-4-en-9-yl)ethanol* (**32**) Under an atmosphere of argon (methoxymethyl)-triphenylphosphonium chloride (17.1 g; 50 mmol) was suspended in anhydrous THF (100 mL) and cooled to 0 °C. Potassium *tert*-butoxide (5.6 g; 50 mmol) was added and the resulting mixture was stirred for 20 min. A solution of **31** (4.95 g, 33.0 mmol) in anhydrous THF (100 mL) was added. The resulting reaction mixture was stirred for 15 min and then poured into a mixture of diethyl ether and water (200 mL/200 mL). The aqueous phase was separated and extracted a second time with diethyl ether (100 mL). The two combined organic layers were dried (Na_2_SO_4_) and concentrated at reduced pressure. The residue was dissolved in THF (200 mL) and aqueous hydrochloric acid (1M, 100 mL) was added. The resulting mixture was heated to reflux for 45 min, cooled to room temperature and poured into a mixture of diethyl ether and water (200 mL/200 mL). The aqueous phase was separated and extracted a second time with diethyl ether (100 mL). The two combined organic layers were dried (Na_2_SO_4_) and concentrated at reduced pressure. The residue was dissolved in methanol (200 mL) and placed under an atmosphere of argon. After cooling the reaction mixture to 0°C, NaBH_4_ (1.89 g; 50 mmol) was added in portions. The mixture was stirred for 15 min, quenched with saturated aqueous ammonium chloride (100 mL) and partitioned between diethyl ether (200 mL) and water (100 mL). The aqueous phase was separated and extracted with diethyl ether (2 × 200 mL). The three combined organic layers were dried (Na_2_SO_4_) and concentrated at reduced pressure. The crude product was purified by column chromatography on silica gel, eluting with a 10–25% gradient of ethylacetate in pentane to provide 4.22 g (77%) of **32**. ^1^H NMR (300 MHz, CDCl_3_) δ 5.70–5.56 (m, 2H), 3.68 (t, *J* = 6.6 Hz, 2H), 2.39–1.94 (m, 6H), 1.51 (q, *J* = 6.7 Hz, 2H), 1.44–1.23 (m, 3H), 0.71–0.57 (m, 2H), 0.30-0.20 (m, 2H).

*Exo-BCN-ethanol* (**33**) A solution of bromine (1.37 mL, 26.7 mmol) in dichloromethane (25 mL) was added dropwise to an ice-cold solution of **32** (4.22 g, 25.4 mmol) in dichloromethane (100 mL). Subsequently, 10% aqueous Na_2_S_2_O_3_ (50 mL) is added. The aqueous phase was separated and extracted a second time with dichloromethane (50 mL). The two combined organic layers were dried (Na_2_SO_4_) and concentrated at reduce pressure to afford dibromide (8.33 g, quant.). Without further purification, the crude intermediate was dissolved in anhydrous THF (100 mL), placed under an argon atmosphere, and cooled to 0°C. A solution of potassium *tert*-butoxide (9.3 g; 83 mmol) in anhydrous THF (100 mL) was added dropwise. The resulting reaction mixture was heated to 70 °C, stirred for 30 min, and quenched with a saturated aqueous solutiono of NH_4_Cl (100 mL). The resulting mixture was extracted twice with diethyl ether (200 mL). The two combined organic layers were then dried (Na_2_SO_4_) and concentrated at reduced pressure. The crude product was purified chromatography on silica gel to afford *exo*-BCN-ethanol (**33**) (2.57 g; 15.6 mmol; 62%) as a slightly yellow solid/wax. ^1^H NMR (300 MHz, CDCl_3_) δ (ppm) 3.71 (t, *J* = 6.5 Hz, 2H), 2.46–2.07 (m, 6H), 1.63–1.54 (m, 2H), 1.44–1.22 (m, 3H), 0.63–0.46 (m, 2H), 0.34–0.22 (m, 1H). ^13^C NMR (300 MHz, CDCl_3_) δ (ppm) 99.6, 63.0, 49.0, 38.6, 34.9, 25.6, 22.8, 22.1. (ESI^+^): calculated for C_11_H_16_O: 164.1201, found 164.1186.

*exo-Bicyclo[6.1.0]non-4-yn-9-ylmethyl (2-(2-(((2-cyanoethoxy)(diisopropyl-amino)phosphino)-oxy)-ethoxy)ethyl)carbamate* (**34**)* Exo*-BCN-ethanol **33** (2.00 g, 12.2 mmol) was dissolved in anhydrous CH_2_Cl_2_ (30 mL) and treated with (*i-*Pr_2_N)_2_POCH_2_CH_2_CN (4.50 mL, 14.1 mmol). A solution of CF_3_CO_2_H (0.25 M) and 1-methylimidazole (0.50 M) in anhydrous CH_2_Cl_2_ (25.0 mL) was added dropwise, then the reaction was stirred at RT for 3 hr. TLC (hexane-acetone, 90:10, PMA stain) shows the complete consumption of *exo*-BCN-ethanol (Rf 0.25) and the appearance of a single new product (Rf 0.70). The reaction mixture was partitioned between CH_2_Cl_2_ (50 mL) and H_2_O (100 mL). The organic layer was washed with H_2_O (100 mL) and saturated NaHCO_3_ (50 mL). The organic layer was dried over Na_2_SO_4, _filtered and concentrated at reduced pressure. Silica (70 g) was slurried in hexane-Et_3_N (95:5, 170 mL) and packed into a 5 cm diameter column. The silica column was eluted with hexanes (80 mL) before a solution of crude product in hexanes (13 mL) was applied. Elution with hexane-acetone (97:3), collecting 50 mL fractions affords purified **34** (3.50 g, 79%) after evaporation of solvent and drying under vacuum for 48 h. ^31^P NMR (500 MHz, CD_3_CN) δ 147.39 (s, 100%). MS (AP+): 365 (M+H); 387 (M+Na).

### HPLC-analysis of **6**, **7** and **8**

The lyophilized ONs were each dissolved in 0.1 M Et_3_N.HOAc-MeCN (95:5, 3 mL) for analysis by reversed phase HPLC analysis: Stationary phase, Supelco 5 M C-18 (150 × 4.6 mm); Mobile phase gradient 10%–35% MeCN in 0.1M TEAA over 25 minutes; Elution 1.0 mL/min; Detection 254 nm.

### SPAAC reaction of **6** and **9**

Oligonucleotide **6** (lyophilized product from a 0.2 mol column) was dissolved in 0.1 M Et_3_N.HOAc/MeCN (90:10, v/v, 0.8 mL). This solution was passed through a 0.2 M PTFE filter into a concentrated solution of **9** (2 mg, 4.8 mol, ~25 equivalents, dissolved in 60 L MeCN). The progress of the SPAAC reaction was monitored by reversed phase HPLC on a C_18_ column (150 × 4.6 mm), eluting at 1.0 mL/min with a gradient of 5%–35% MeCN in 0.1 M Et_3_N.HOAc over 30 min, recording UV absorption at 260 nM. Excess **9** does not absorb at this wavelength and, therefore, only **6** and **10** are visible in the chromatograms. Mass spectral analysis of the major peak corroborated the expected formation of product **10**. MS (AP+): 2521.2 (M+H).

*5′-O-(4,4′-Dimethoxytriphenylmethyl)-2′-O-(9H-fluoren-9-yl-methylcarbonyl)-aminoprop-1-yl-6-N-benzoyladenosine* (**12**) To the solution of 5′-*O*-(4,4′-dimethoxytriphenylmethyl)-2′-*O*-azidoprop-1-yl-6-*N*-benzoyladenosine **11** (750 mg, 0.99 mmol) in THF/H_2_O (2:1) (18 mL), trimethylphosphine (1.5 mL, 1.5 mmol) was added. Reaction stirred at rt for 8 h and evaporated to dryness. The residue was taken into Dioxane/H_2_O (1:1) (20 mL), NaHCO_3_ (185 mg, 2.2 mmol) was added. The reaction mixture was cooled down to 0 °C, Fmoc-OSu (415 mg, 1.23 mmol) in dioxane (2 mL) was added drop wise and stirred for 15 min at 0 °C. The reaction was quenched with water, extracted with CH_2_Cl_2_ (3 × 20 mL), the combined organic layer was dried over Na_2_SO_4_ and concentrated to dryness. Flash-chromatography (CH_2_Cl_2_/acetone, 6:4, v/v), gave the desired compound (850 mg, 90%) as a colorless foam. ^1^H-NMR (400 MHz, DMSO) δ 11.23 (s, 1H), 8.68 (s, 1H), 8.62 (s, 1H), 8.06 (d, *J* = 7.2 Hz, 2H), 7.87 (d, *J* = 7.2 Hz, 2H), 7.66 (m, 3H), 7.57 (m, 2H), 7.39–7.18 (m, 14H), 6.85 (m, 4H), 6.19 (d, *J* = 4.8 Hz, 1H), 4.65 (m, 1H), 4.48 (m, 1H), 4.28 (d, *J* = 6.8 Hz, 2H), 3.71 (s, 3H), 3.65–3.53 (m, 2H), 3.27 (m, 2H), 3.26 (m, 2H), 1.66 (m, 2H). ^13^C-NMR (75 MHz, DMSO) δ 165.7, 158.0, 157.8, 156.1, 151.9, 151.6, 150.5, 144.8, 143.9, 143.2, 140.7, 135.6, 135.4, 133.4, 132.4, 129.7, 128.9, 128.5, 128.5, 127.8, 127.7, 127.5, 127.4, 127.0, 126.6, 125.8, 125.0, 120.1, 113.1, 112.8, 86.3, 85.5, 83.6, 80.3, 69.1, 67.7, 65.2, 63.5, 55.0, 46.8, 37.3, 29.6. (ESI^+^): calculated for C_56_H_52_N_6_O_9_ (M+H^+^): 953.3874, found 953.3866.

*5′-O-(4,4′-Dimethoxytriphenylmethyl)-2′-O-(9H-fluoren-9-yl-methylcarbonyl)-aminoprop-1-yl-6-N-benzoyladenosine-3′-O-(2-cyanoethyl N,N-diisopropylphosphoramidite)* (**13**) Compound **12** (120 mg, 0.126 mmol) dried overnight at high vacuum, was dissolved in CH_2_Cl_2_ (2 mL) and flushed with N_2_ for 5 min. DIPEA (29 µL, 0.166 mmol) was added to the reaction mixture followed by (*i*-Pr_2_N)P(OCH_2_CH_2_CN)Cl (37 µL, 0,166 mmol). The reaction was stirred for 1 h, at rt, under N_2_ atmosphere. It was diluted with CH_2_Cl_2_ (20 mL) and 2.5% aqueous NaHCO_3_ solution (10 mL) was added. Organic layer was separated and aqueous layer was extracted with CH_2_Cl_2 _(2 × 10 mL). The combined organic layers were dried over Na_2_SO_4_, filtered and concentrated in vacuo. The residue was purified by flash chromatography (CH_2_Cl_2_/acetone, 8:2) to give the desired compound **13** (850 mg, 90%) as colorless foam. ^1^H-NMR (500 MHz, CDCl_3_) δ 8.91 (s), 8.72 (d), 8.26 (s), 8.18 (s), 7.93 (d), 7.66 (d), 7.61–7.20 (m), 6.79 (dd), 6.17 (d) 5.22 (br s), 4.74–4.62 (m), 4.44–4.28 (m), 4.20 (t), 3.78 (d), 3.76–3.44 (m), 3.40–3.18 (m), 2.47 (t), 2.30 (t), 1.77 (br s), 1.19–1.05 (m). ^31^P NMR (500 MHz, CDCl_3_) δ 150.19, 149.84. HRMS (ESI^+^): calculated for C_65_H_69_N_8_O_10_P (M+H^+^): 1153.4918, found 1153.4952.

### Synthesis of dinucleotides **16**–**21**

*Dinucleotide-CPG (Fmoc-on)* (**14**)***.***Solutions of **13** (140 mg, 0.12 mmol) in MeCN (1 mL) and DCI (450 mg, 3.6 mmol) in MeCN (5.5 mL) were stored separately over 4 Å MS for 20 min, then mixed together and introduced into the cartridge containing thymidine-CPG (300 mg). The coupling reaction was performed for 25 min with manual mixing. The CPG was washed with MeCN (3 × 5 mL) and dried. To which oxidizing agent (4% iodine in THF/pyridine/H_2_O) (2 mL) was added and mixed manually for 30 min. The resulting CPG was washed with DMF (2 × 5 mL), MeCN (2 × 5 mL) and dried to give dinucleotide CPG (325 mg).

*Dinucleotide-CPG (Fmoc-off)* (**15**). Dinucleotide-CPG **14** (300 mg) was treated with piperidine (20% in DMF) (1 mL) for 20 min at rt. It was washed with DMF (2 × 1 mL), CH_2_Cl_2_ (2 × 1 mL) and dried to give Fmoc-off CPG **15** (250 mg). 

*Dinucleotide (Fmoc-off)* (**16**). Fmoc-off CPG 1**5** (20 mg) was treated with aq. NH_4_OH solution (0.5 mL) for 12 h, 55 °C. The solution was filtered-off and dinucleotide **16** was confirmed by HPLC and HR-MS analysis.

### General procedure for the synthesis of **17**–**21**

A solution of corresponding carboxylic acid (0.019 mmol), HATU (0.019 mmol) and NMM (5 L) in DMF (200 L), was added to the dried Fmoc-off CPG **15**. The resulting mixture was stirred for 2 h at rt and filtered off. It was further washed with DMF (2 × 1 mL), MeCN (2 × 1 mL), CH_2_Cl_2_ (2 × 1 mL) and dried. The corresponding coupled dinucleotides **17**–**21** were cleaved from CPG with aq. NH_4_OH solution (0.5 mL) for 12 h, 55 °C and confirmed by HPLC and HR-MS analysis.

For **21a** (DMT off). A solution of azidoacetic acid (0.019 mmol), HATU (0.019 mmol) and NMM (5 L) in DMF (200 L), was added to the dried Fmoc-off CPG **15**. The resulting mixture was stirred for 2 h at rt and filtered off. It was further washed with DMF (2 × 1 mL), CH_2_Cl_2_ (2 × 1 mL) and treated with 1.5% TFA in CH_2_Cl_2_ for 2 min. Removal of solvent, washing of CPG with CH_2_Cl_2_ (2 × 1 mL) and subsequent cleavage with aq. NH_4_OH solution (0.5 mL) for 12 h, 55 °C, afforded the azido dinucleotide (5′-OH) **21a**, as confirmed by HPLC and HR-MS analysis.

### SPAAC reactions and analysis of **20** with **21** and **22**

The solutions of **20** (50 mL) and **21** (100 mL**)** or **22** (100 mL, 1 mg/100 mL) in water were mixed for 2 min to afford the (3+2) cycloaddition product, as confirmed by HPLC and HR-MS analysis.

### Synthesis and conjugation of dodecamer ONs **25** and **26**

CPG **23** (50 mg) was treated with piperidine (20% in DMF) (1 mL) for 25 min at rt. It was washed with DMF (2 × 1 mL), CH_2_Cl_2_ (2 × 1 mL) and dried to give Fmoc-off CPG **24** (48 mg). A solution of activated BCN-hydroxysuccinimide carbonate or azidoacid hydroxysuccinimide ester (0.019 mmol) in DMF (100 L) was mixed with CPG **24** (20 mg), to which DIPEA (0.029 mmol) was added. The resulting mixture was stirred for 12 h at rt and filtered off. It was further washed with DMF (2 × 1 mL), MeCN (2 × 1 mL), CH_2_Cl_2_ (2 × 1 mL) and dried. The corresponding ON-conjugates were cleaved from CPG with aq. NH_4_OH solution (0.5 mL) for 12 h, 55 °C. Purification on Sep-pak cartridge afforded the ON-conjugates **25** or **26**, respectively, as confirmed by HPLC and MALDI-TOF analysis.

### Dimerization of **25** and **26** to give **27**

The solutions of **25** (50 L, 28 nmol) and **26** (70 L, 32 nmol) in water were mixed for 20 min to afford the (3+2) cycloaddition product **27**, as confirmed by HPLC (Figure S3) and MALDI-TOF analysis.

### Preparation of **28**

Bromoterminated poly(3-hexyl thiophene) (M_n_ = 3000, PDI = 1.35) was reacted with excess amount of sodium azide in CH_2_Cl_2_ overnight. After precipitation in MeOH and drying, azide-terminated P3HT **28** was obtained as product. FT-IR (cm^−^^1^): 3052 (γ_-C=CH, aromatic_), 2952-2854 (γ_-C-H aliphatic_) 2085 (γ_-N3_), 1509 (γ_-C=C, asym ring_), 1456 (γ_-C=C, sym ring_), 1376 (γ_-CH3, deformation_), 821 (γ_-C-H, aromatic, out-of-plane_) and 725 (γ_-CH2, rocking_).

### Conjugation of **26** to polymer **28**

Solution of P3HT-azide **28** (1mg /10 mL, in CH_2_Cl_2_) was stirred with aqueous slution of BCN conjugated ON **26** for 24 h at room temperature to get P3HT-ON bioconjugate. Aqueous layer treated several times with DCM to wash out unreacted P3HT.

### Preparation of **29**

Solution of P3HT-azide **28** (1 mg/10 mL, in CH_2_Cl_2_) was stirred with aqueous slution of BCN conjugated ON **26** for 24 h at room temperature to get P3HT-ON bioconjugate. Aqueous layer treated several times with DCM to wash out unreacted P3HT.

## 4. Conclusions

We have successfully demonstrated the suitability of BCN and derivatives thereof for the preparation of oligonucleotide conjugates by (3+2) cycloaddition with azides. Several BCN-derived phosphoramidites were prepared and incorporated into oligonucleotides with high yield and purity. In addition, an Fmoc-protected 2′-modified adenosine derivative, prepared in only 6 steps from adenosine, served as a versatile building block for both solution and solid phase ON synthesis, thereby allowing the introduction of a functional group at any adenosine during or after oligonucleotide synthesis, and potential extension to other nucleobases (research in progress). The BCN-containing oligonucleotides were found to undergo fast SPAAC functionalization or dimerization, and were even suitable for conjugation to lipophilic polymers. The coupling of oligonucleotide to azide-substituted polythiophene opens the possiblity for the construction of a variety of simple field effect transistor biodevices in which sequence specific ON functionalized conjugated polymers are the key component. In a broader context, we have demonstrated the unique combination of reaction efficiency and selectivity of cyclooctyne-based chemistry for the conjugation of sensitive (bio)molecules in aqueous systems, which may be readily extended toward the conjugation of BCN-oligonucleotides to azide-containing solid surfaces, polymers and large proteins. Finally, we [39] and others [40,41] recently demonstrated that cycloadditions of BCN is not limited to azides, but BCN also undergoes extremely fast strain-promoted inverse-electron-demand Diels-Alder cycloaddition (SPIEDAC) with tetrazines. In contrast, benzofused cyclooctynes DBCO and DIBO are inreactive towards tetrazine [42], which further lifts the potential of BCN-modified oligonucleotides for fast and selective bioconjugations, potentially also *in vivo* [43]. Research along this line, as well as extension of the strategy towards other 2′-O-alkylated nucleobases, is currently ongoing in our laboratory.

## Supplementary Materials

Supplementary materials can be accessed at: http://www.mdpi.com/1420-3049/18/7/7346/s1.
