# Supplementary material for: Oligonucleotide Tagging for Copper-Free Click Conjugation
Source: Molecules. 2013 Jun 24;18(7):7346–63. doi: 10.3390/molecules18077346 (PMC6270431; doi:10.3390/molecules18077346)

Supplementary Materials

Figure S1. HPLC-traces for conversion of **6** to **10**.

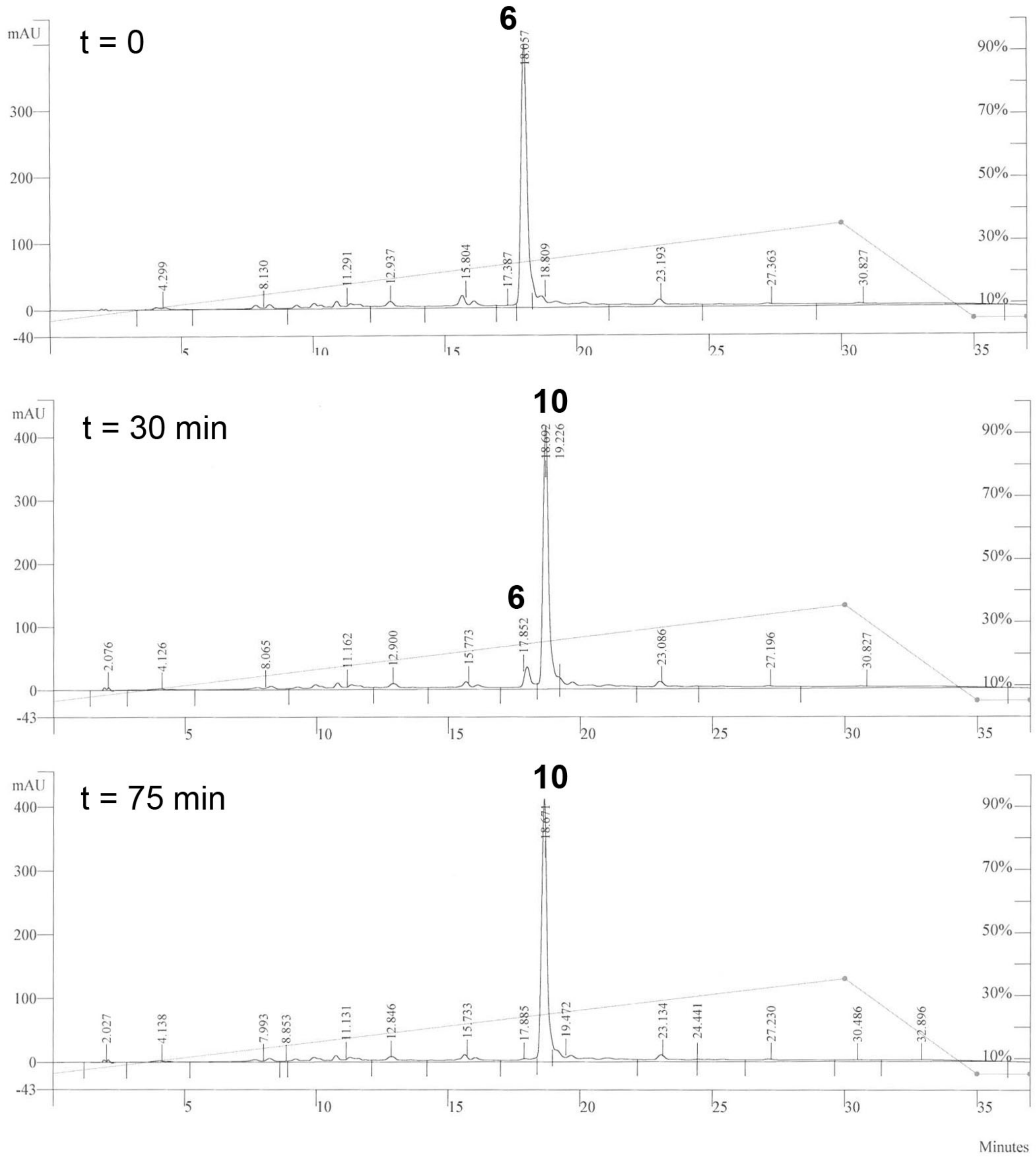

HPLC method

Dinucleotides:

RP-18 column; gradients of H_2O (A) and MeCN (B): 35 min 0-30% B in A, flow rate 1 mL/min.

Oligonucleotides:

RP-18 column; gradients of 0.1 M (Et_3NH)OAc (pH 7.0)/MeCN 95 :5 (A) and MeCN (B): 40 min 0–50% B in A, flow rate 1 mL/min;

Table S1. HR-MS Analysis of compounds **16–21** and cycloadducts.

Entry	Compound	$(M+H^+)$ Calculated	$(M+H^+)$ Found
1	**16**	931.3481	931.3391
2	**17**	1178.4641	1178.4600
3	**18**	1157.4177	1157.4167
4	**19**	1289.3906	1289.3869
5	**20**	1107.4278	1107.4228
6	**21**	1012.3406	1012.3355
7	**21a** (DMT off)	712.2198	712.2204
8	cycloaddition adduct (**20+21a**)	1816.6159	1816.6198
9	cycloaddition adduct (**20+22**)	1281.5403	1281.5345

Dimerization of 25 and 26 to give 27

Table S2. MALDI-TOF data for compounds **25–27**.

Entry	Compound	MW Calculated	MW Found
1	**25**	3855.401	3856.201
2	**26**	3893.125	3894.145
3	**27**	7748.50	7748.776

Figure S2. HPLC-profiles of **16–21**, conjugation with **22** and dimerization of **20** and **21**.

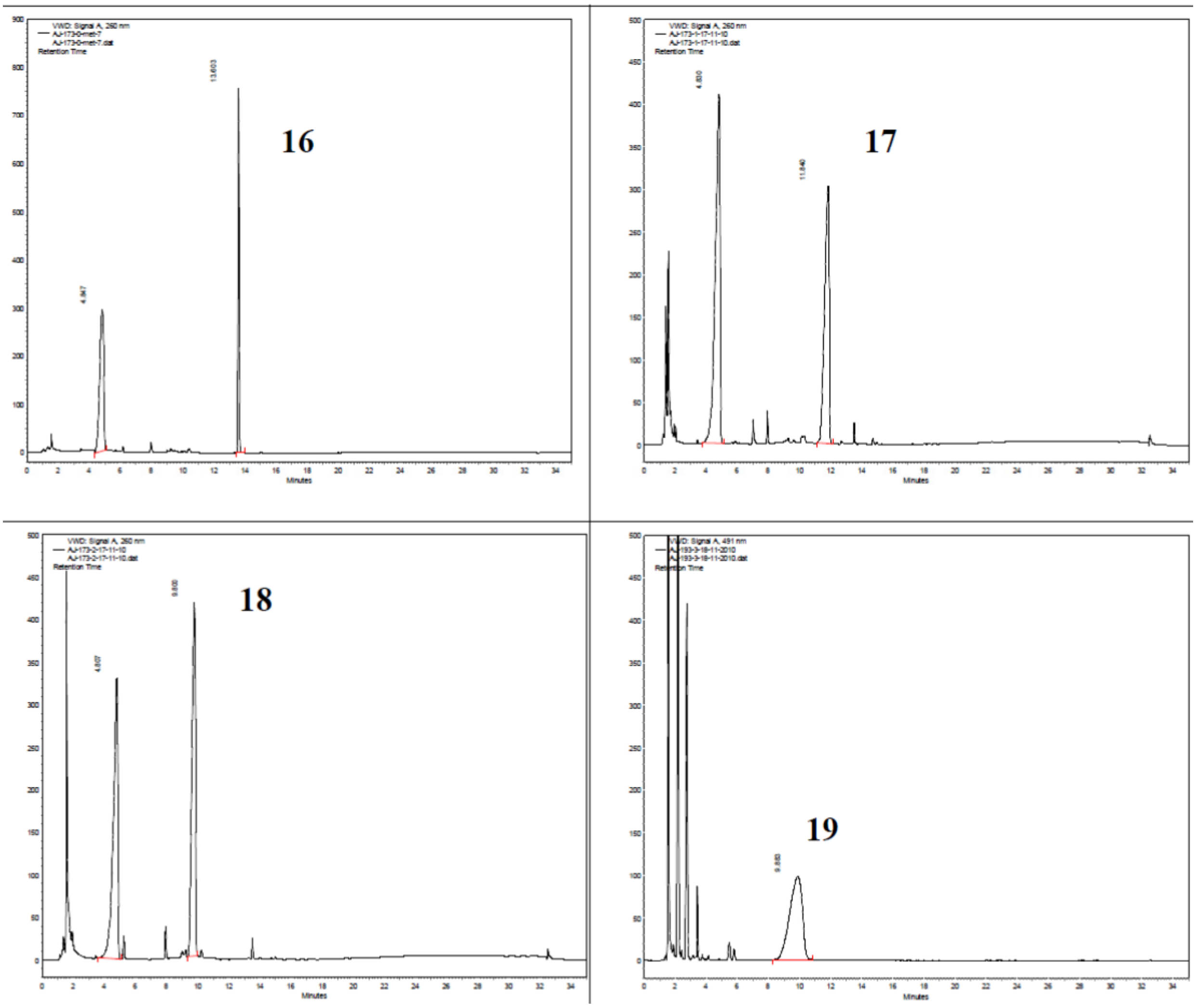

Figure S2. *Cont.*

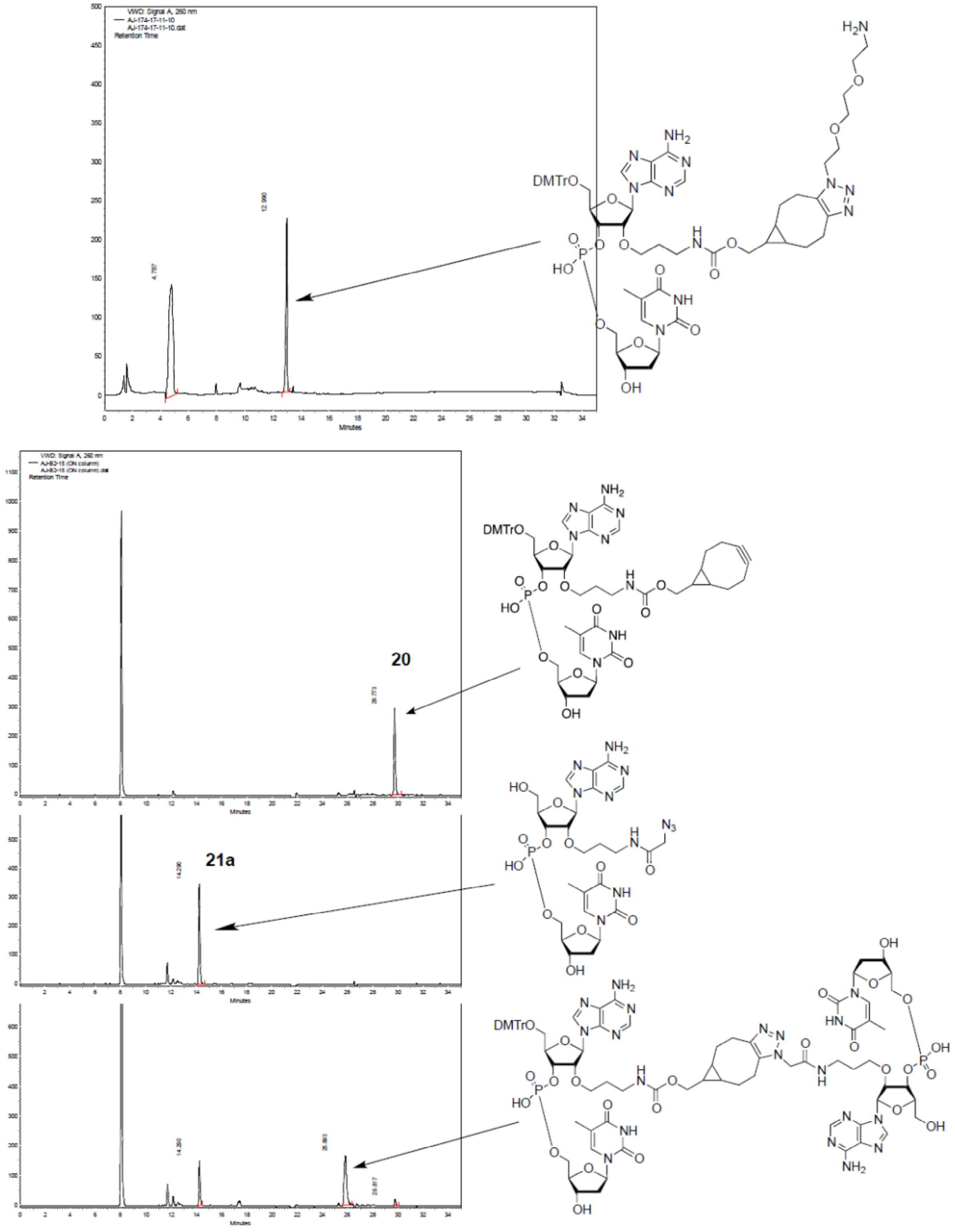

Only numbered peaks are dinucleotide conjugates. The rest of the peaks result from deprotection (by ammonia) of protecting group benzoyl of adenosine, beta-elimination of -OCH_2CH_2CN and 3'-CPG-linker residue. In all above cases, dinucleotide-Fmoc was deprotected on resin, then washed and immediately followed by conjugation. After that, the dinucleotide conjugates were cleaved from resin by ammonia and samples were directly injected on HPLC.

Formation of conjugates was confirmed by HR-MS.

Table S3. HPLC retention times of **16–21**, conjugation of **20** with **22** and dimerization of **20** and **21**.

Compound	Retention time (min)
**16**	13.60
**17**	11.84
**18**	9.8
**19**	9.88
conjugation of **20** with **22**	12.99
**20**	29.77
**21**	14.29
dimerization of **20** and **21**	25.89

Figure S3. HPLC-profile of SPAAC dimerization of **25** and **26** leading to **27**.

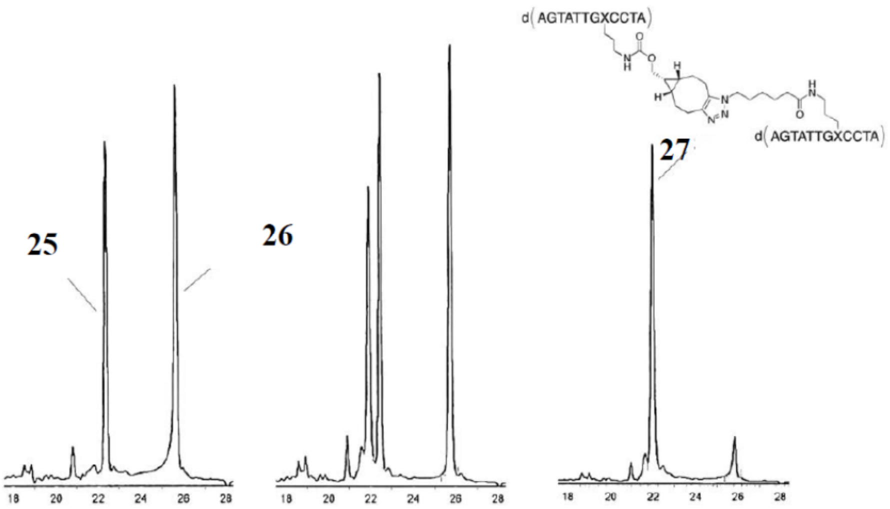

Figure S4. MALDI-TOF results: (**a**) blue line for ON **26** and (**b**) green line for P3HT/ON composite **29**.

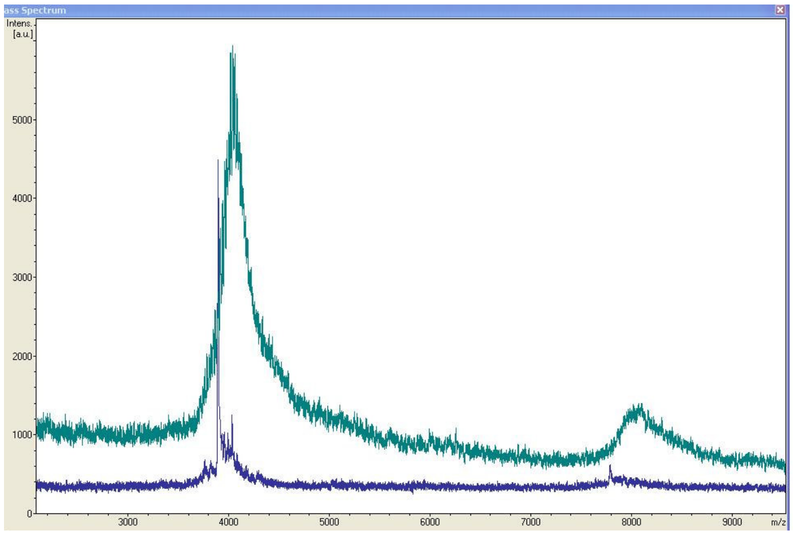

Molecular weight of ON **26** is 3957 D.

Molecular weight of P3HT (average) is 4292 D (not shown).

Calculated mass of P3HT/ON composite **29** is 8049 D, observed mass of major peak is 8060 D in linear mode. As P3HT is polymer, there is a distribution of polymer chains, which remains after conjugation with ON **26**.

Figure S5. Compound **1** - ^{1}H-NMR (CD_3CN).

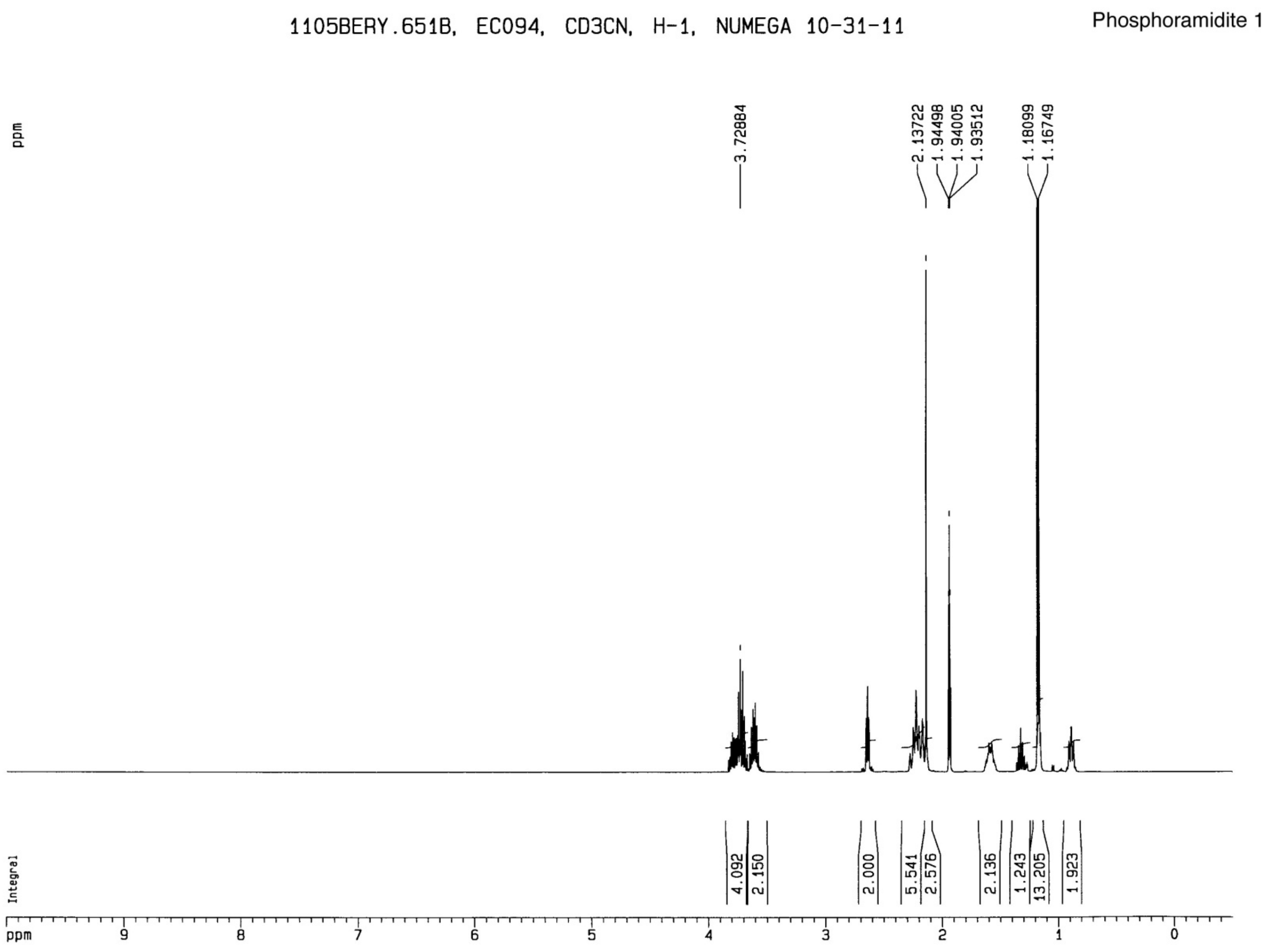

Figure S6. Compound **1** - ^{31}P-NMR (CD_3CN)

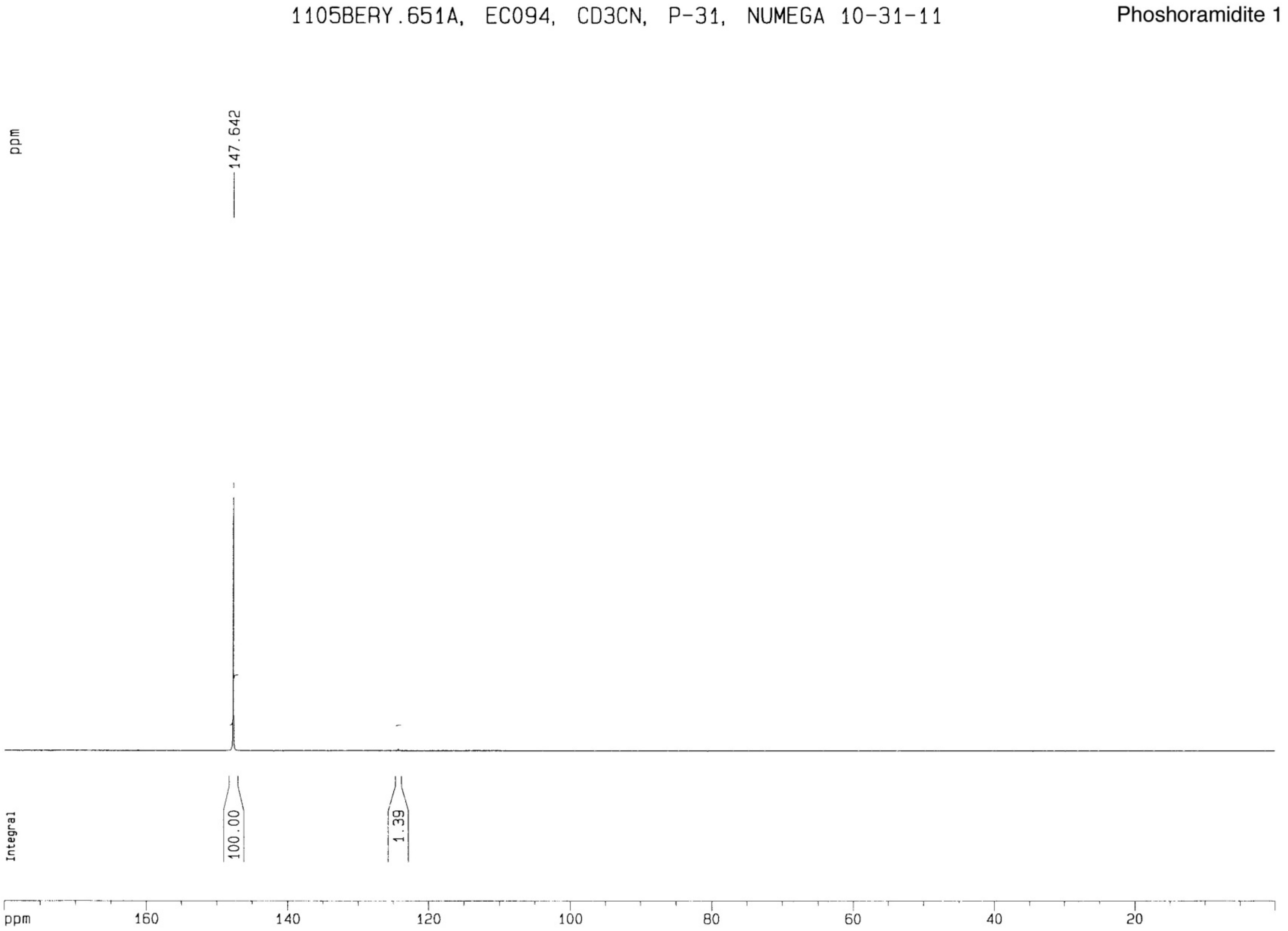

Figure S7. Compound **2** - ^{1}H-NMR (CD_3CN).

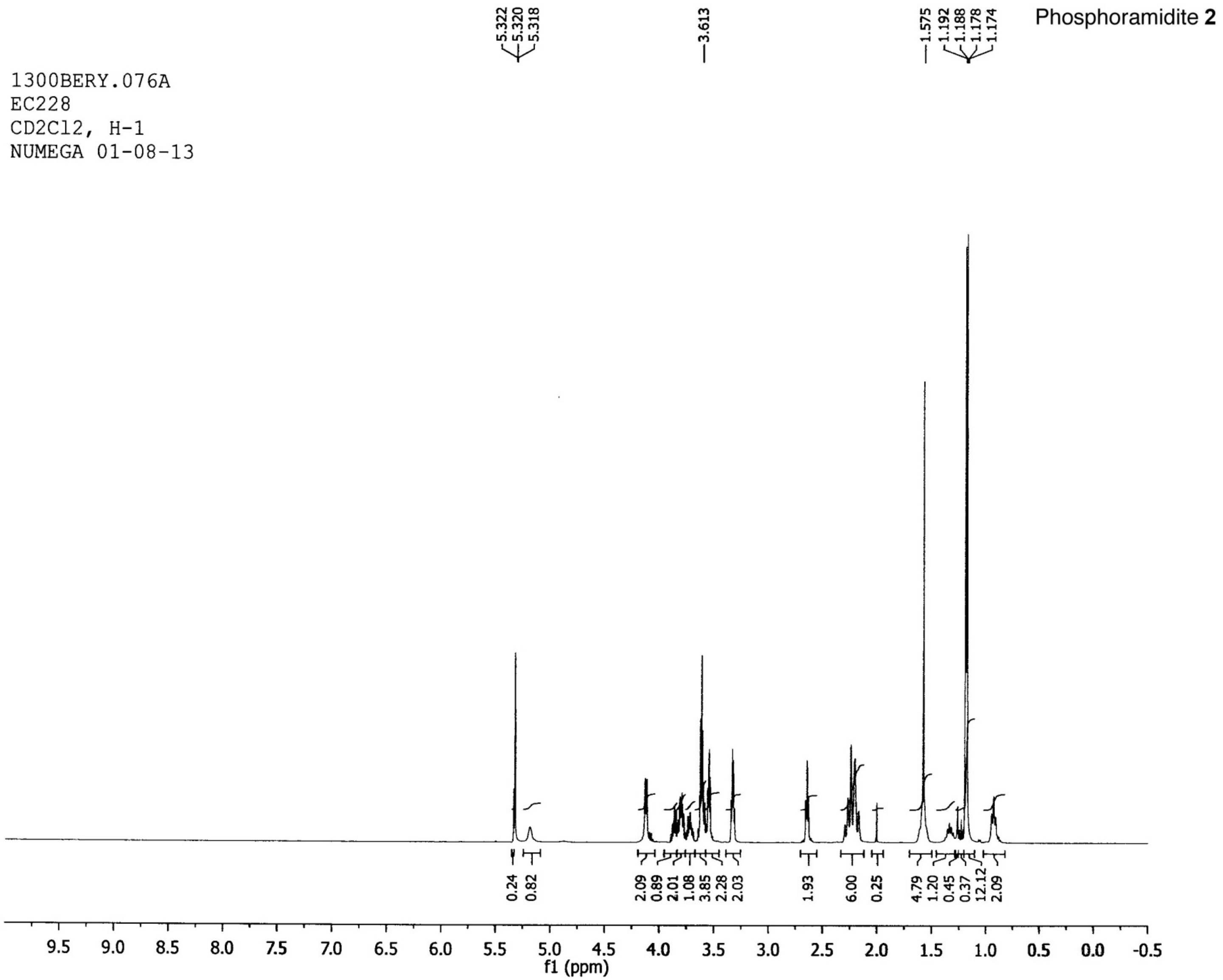

Figure S8. Compound **2** - ^{31}P-NMR (CD_3CN).

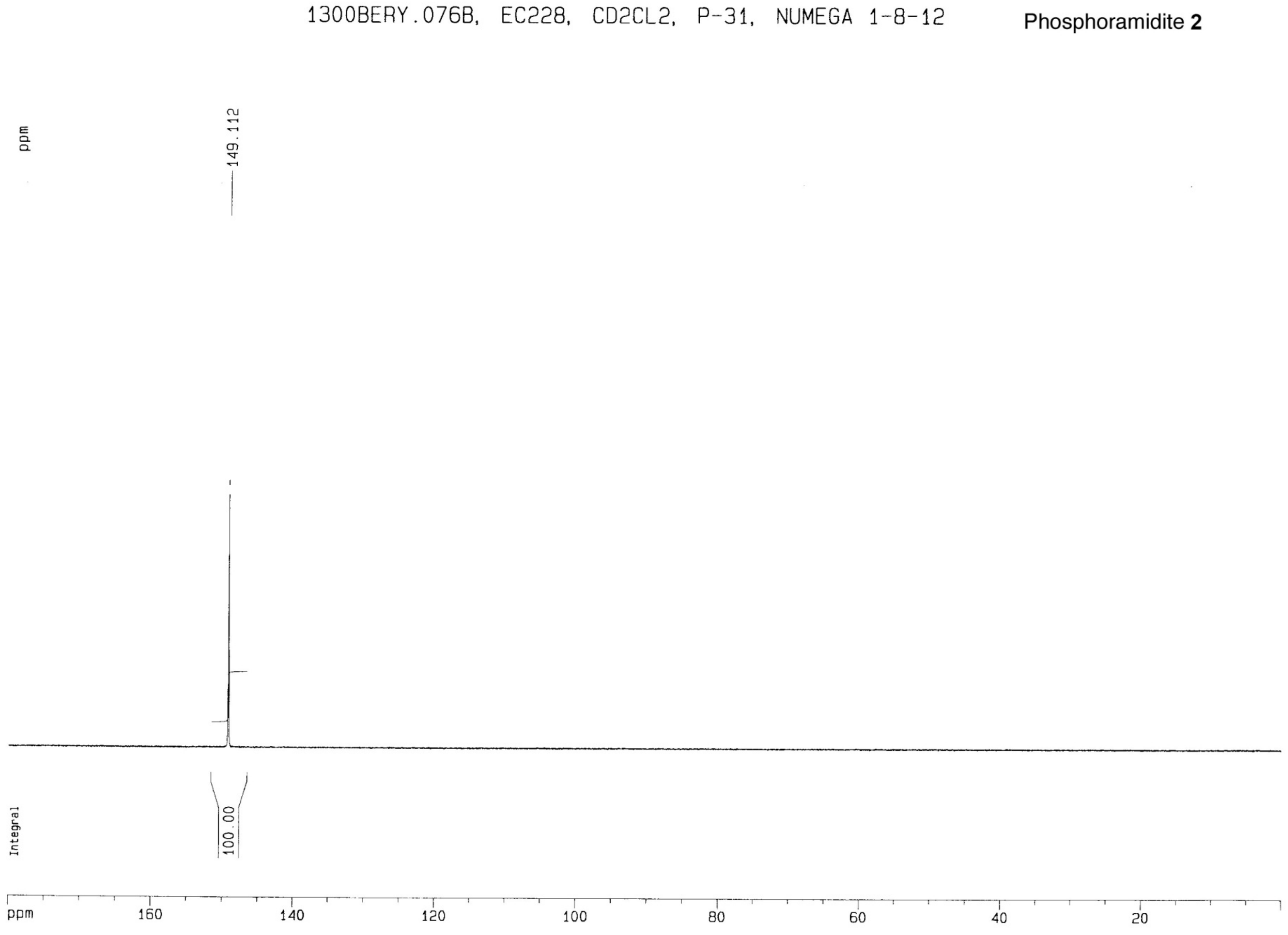

Figure S9. Compound **31** - ^{1}H-NMR.

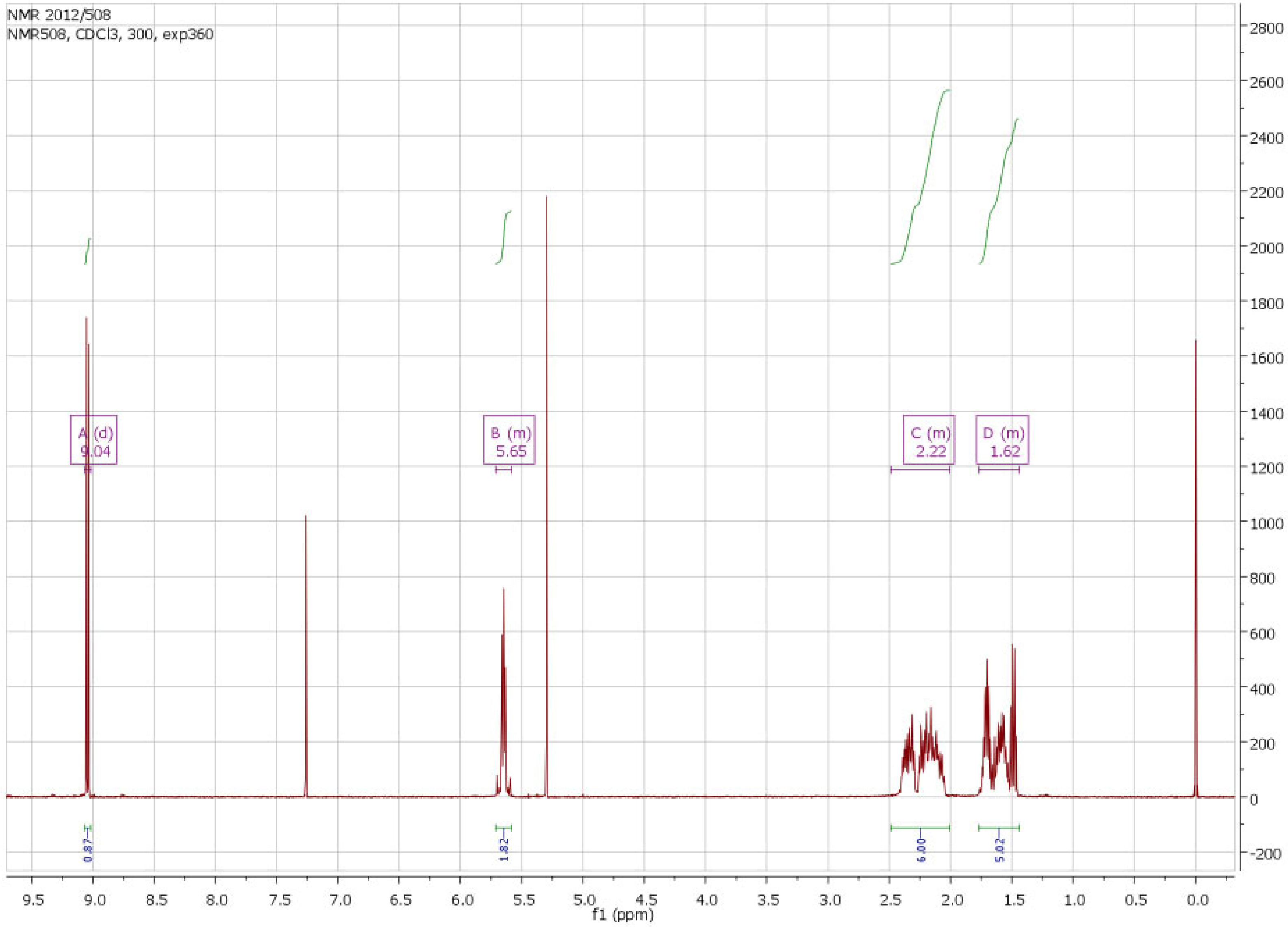

Figure S10. Compound **32** - ^{1}H-NMR.

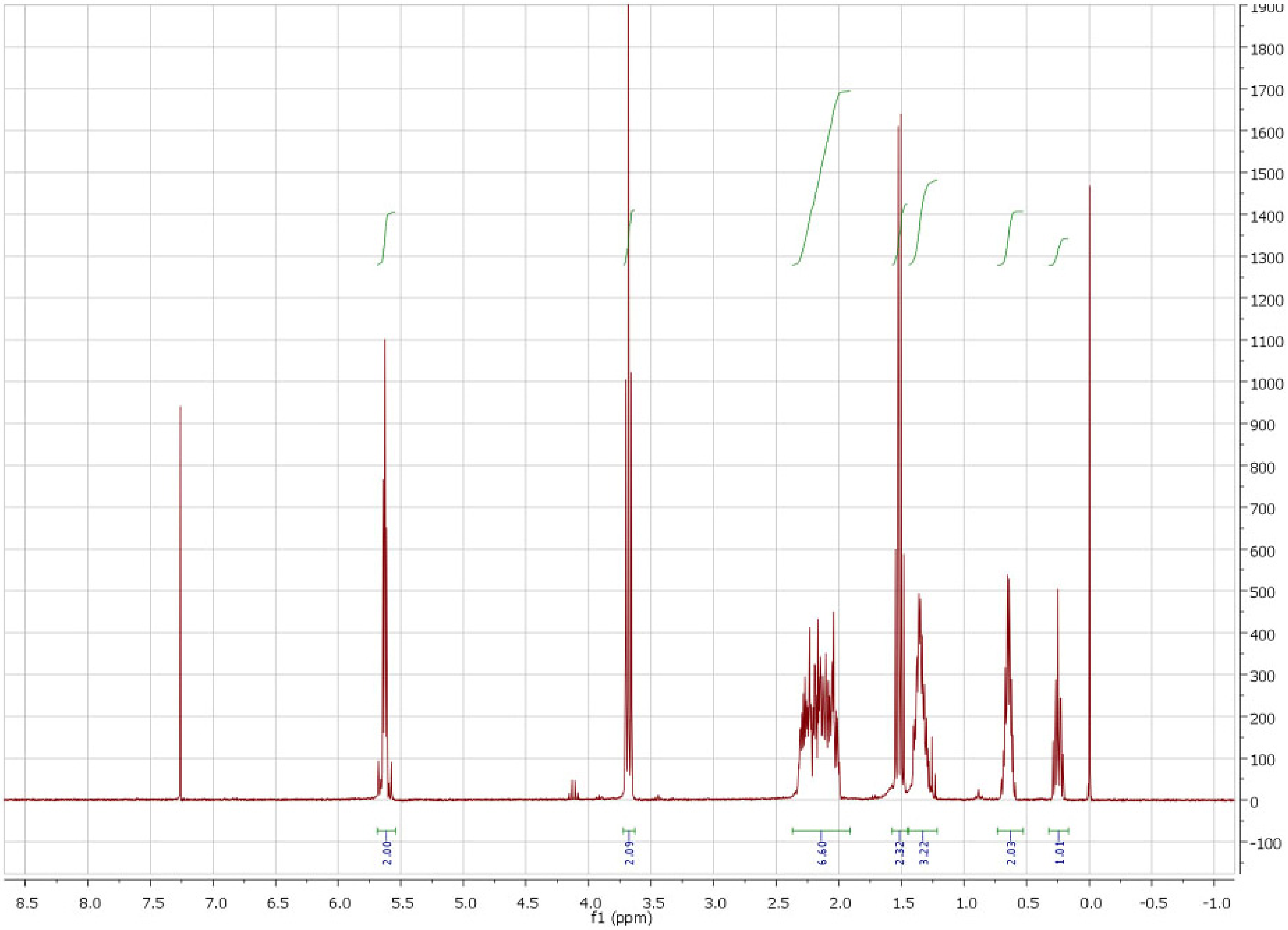

Figure S11. Compound **33** - ^{1}H-NMR.

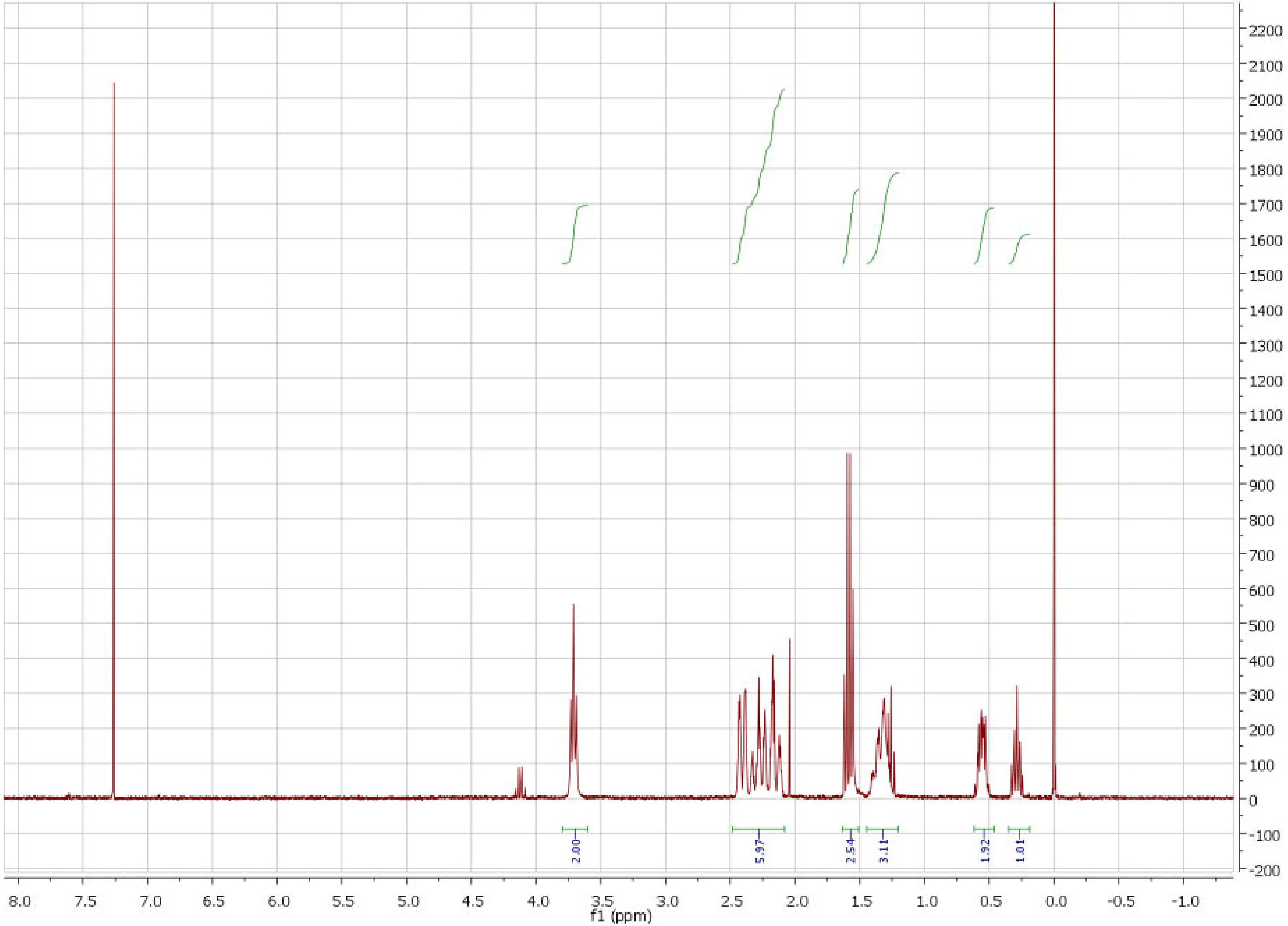

Figure S12. Compound **33** - ^{13}C-NMR.

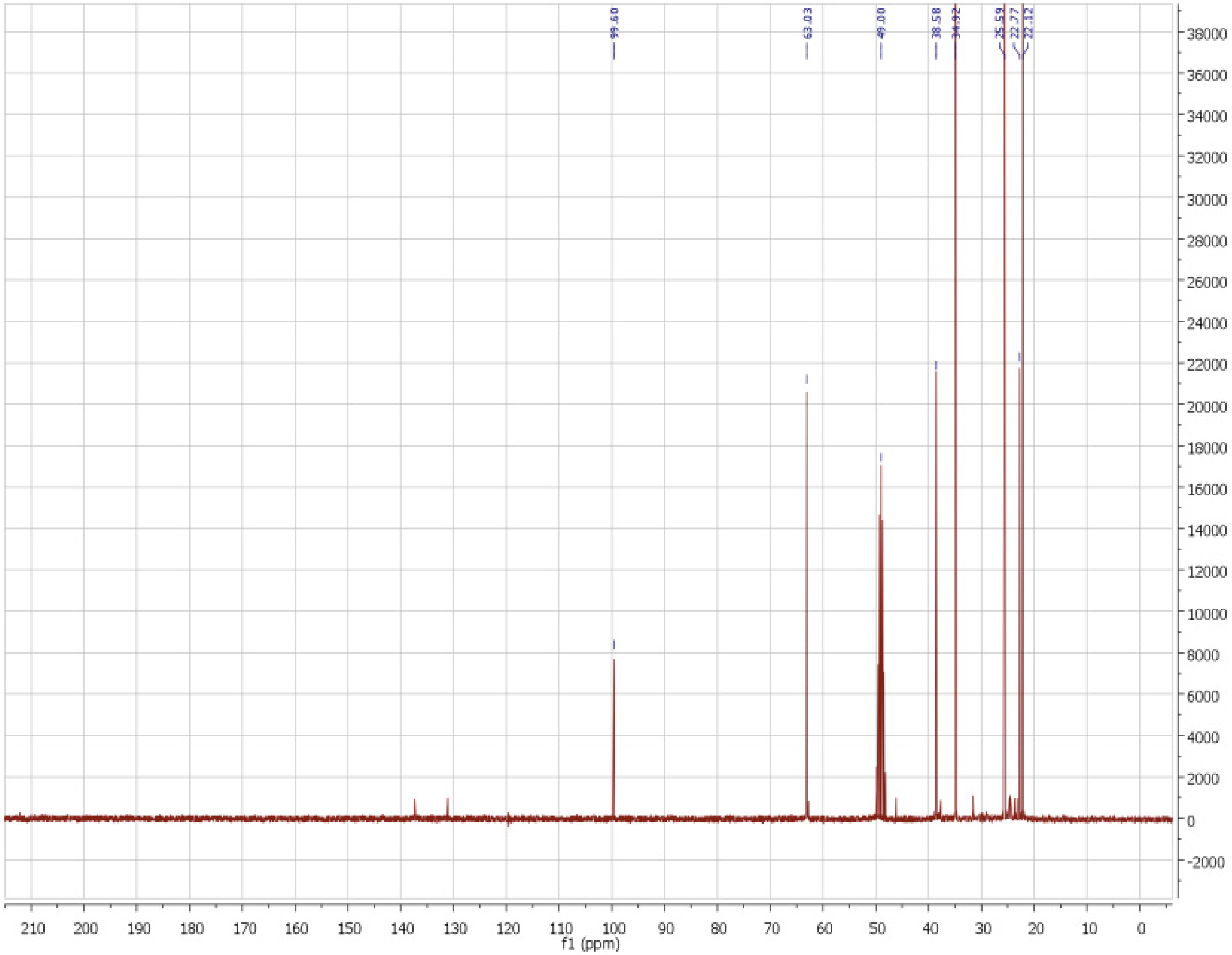

Figure S13. Compound **34** - ^{1}H-NMR (CD_3CN).

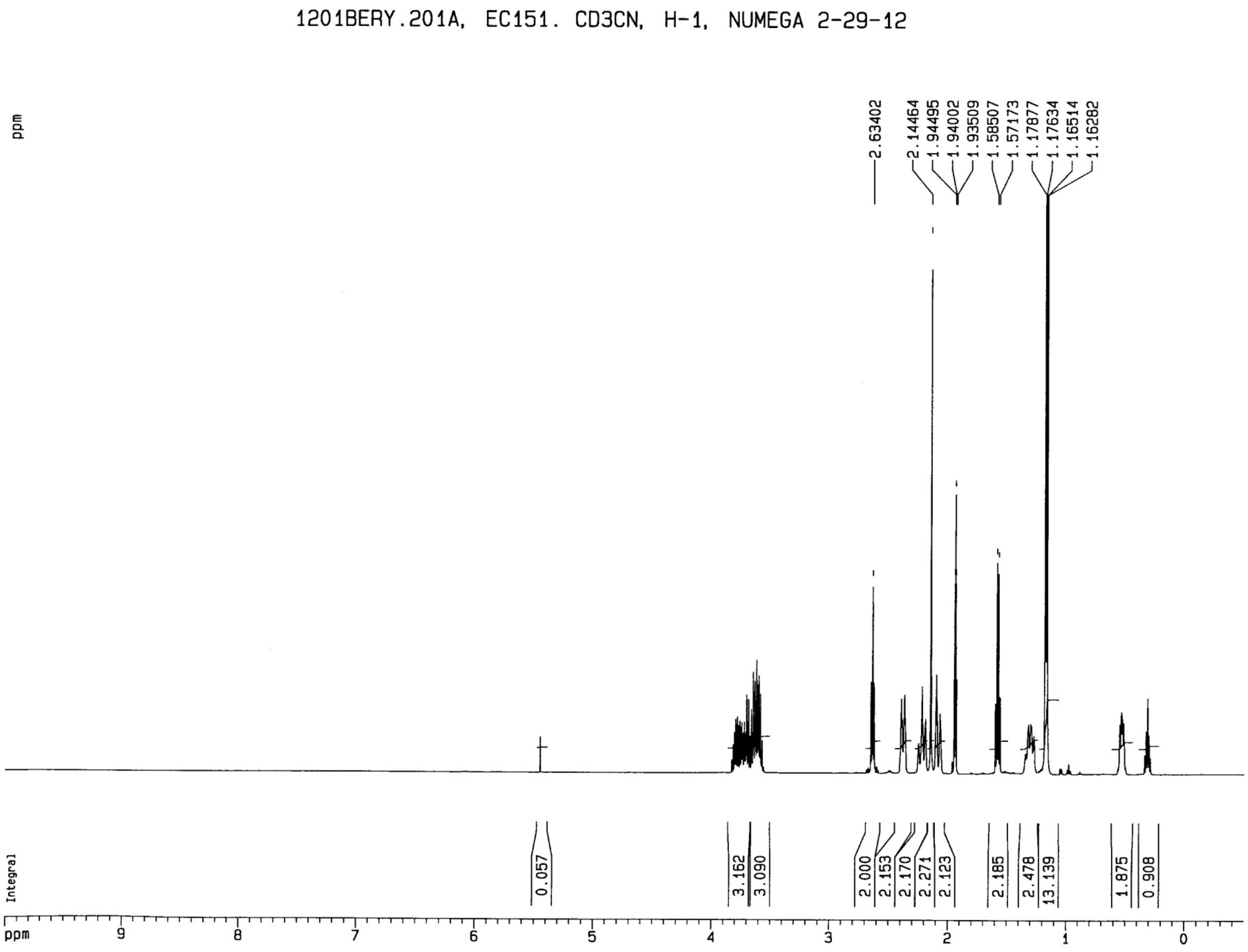

Figure S14. Compound **34** - ^{31}P-NMR (CD_3CN).

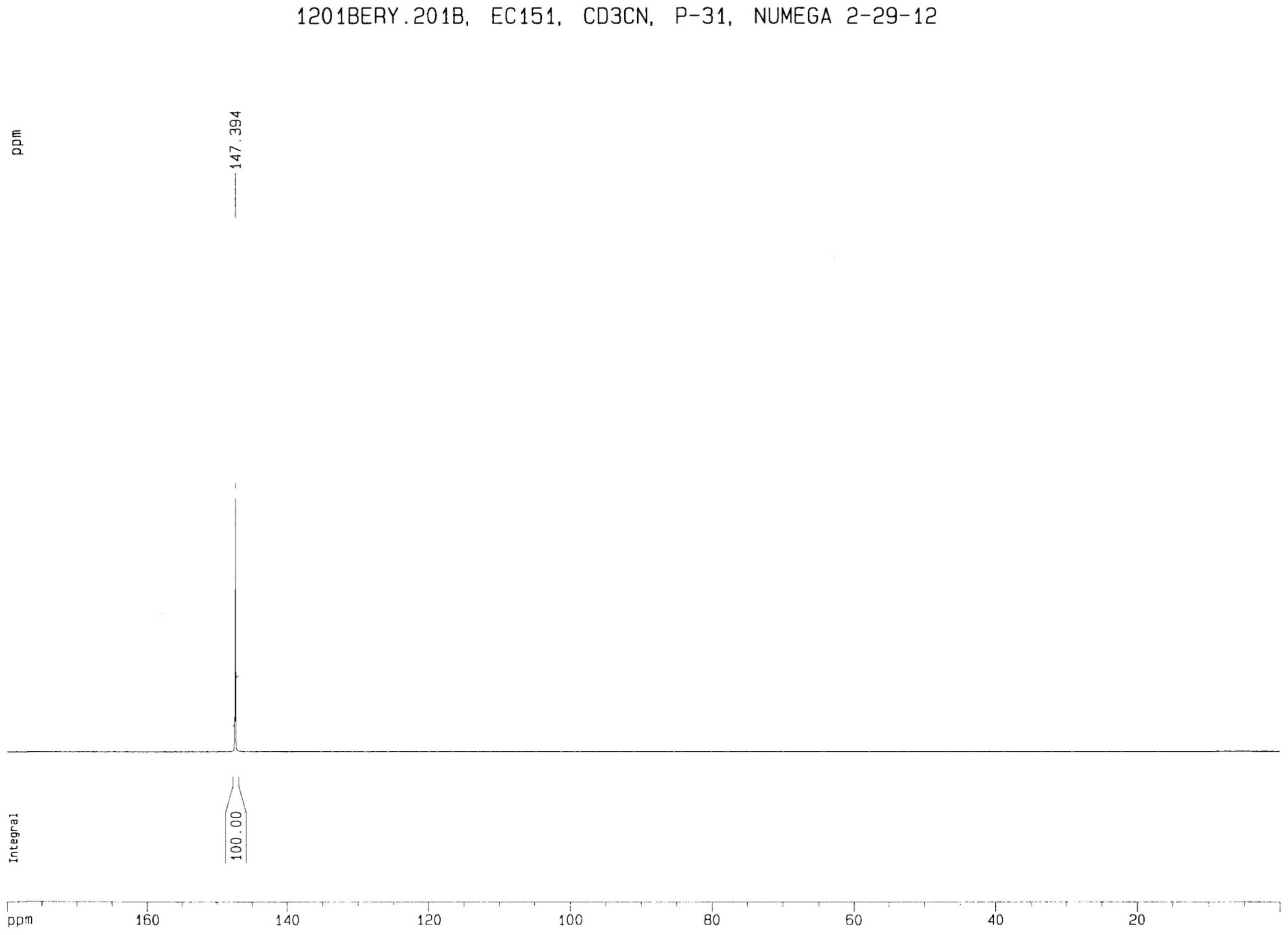

Figure S15. Compound **12** - ^{1}H-NMR.

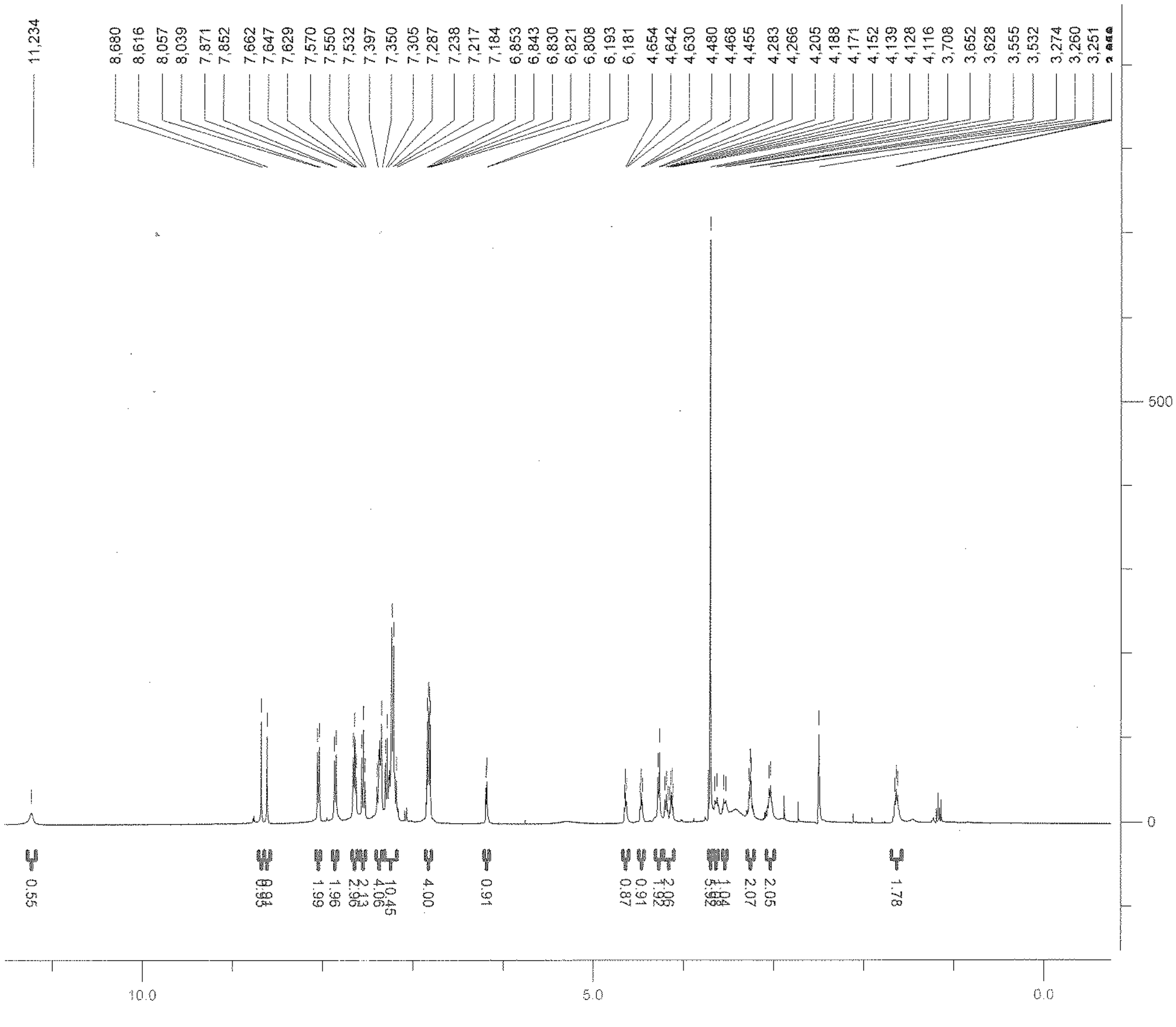

Figure S16. Compound **12** - ^{13}C-NMR.

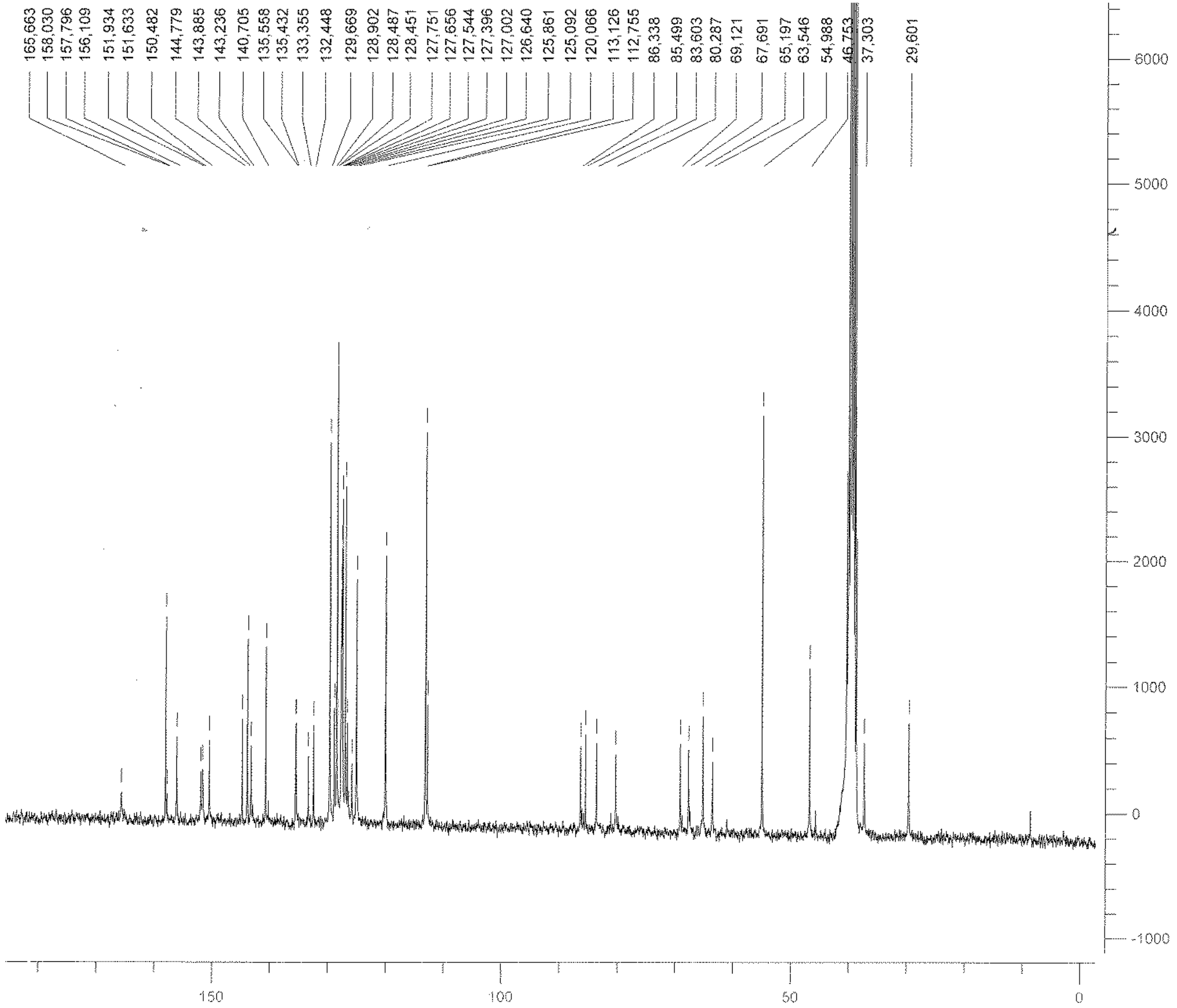

Figure S17. Compound **13** - ^{1}H-NMR.

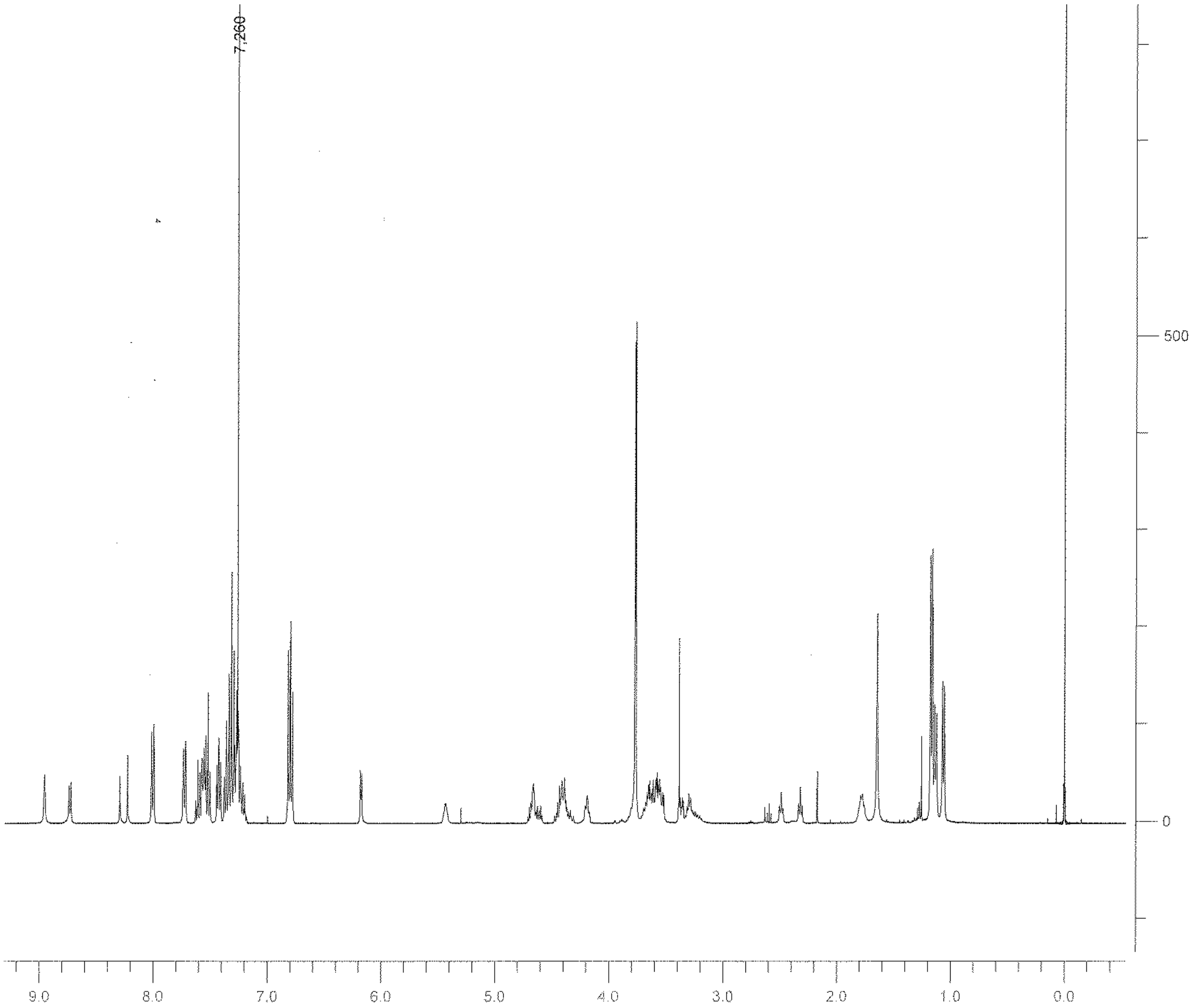

Figure S18. Compound **13** - ^{31}P-NMR

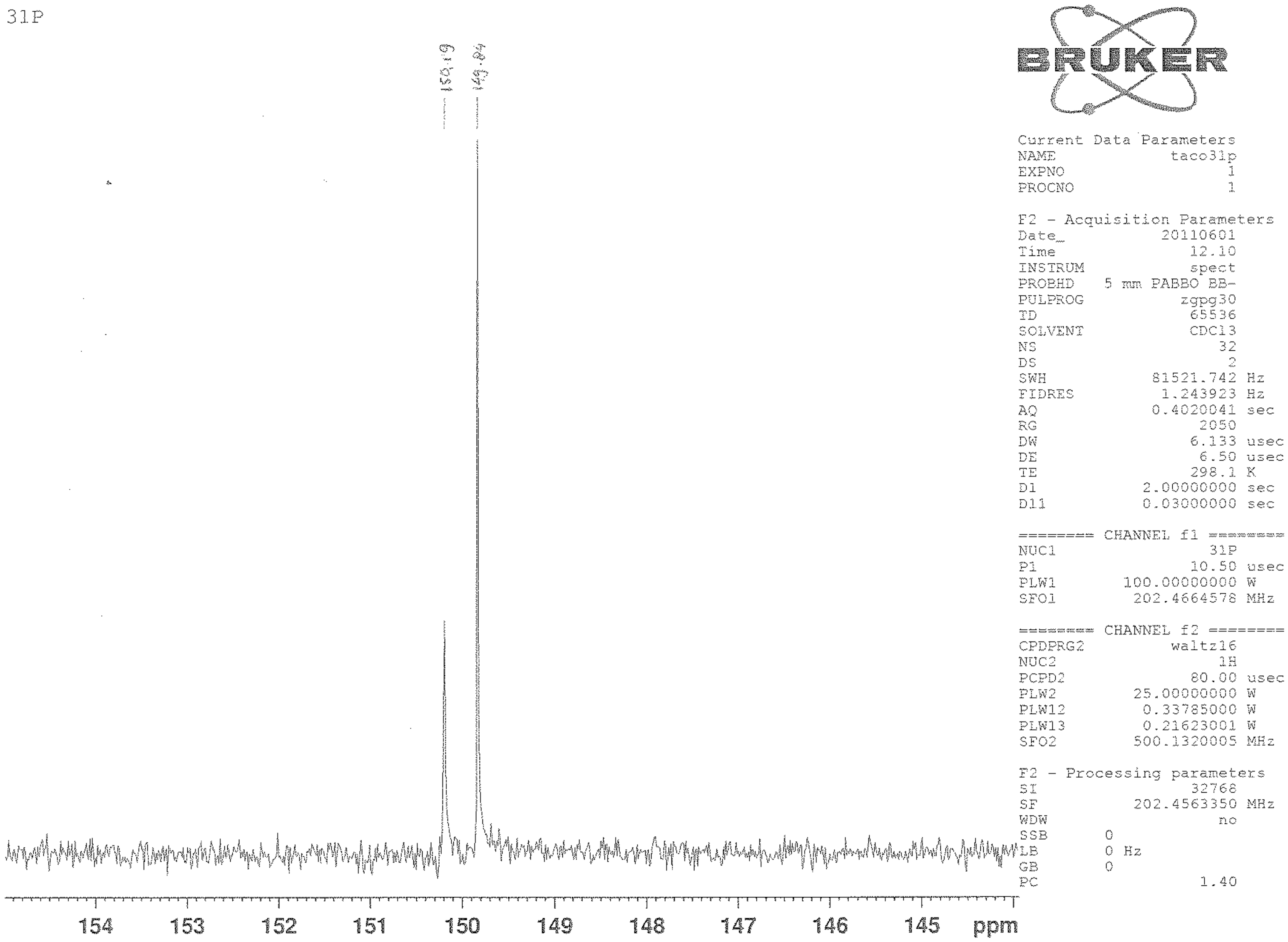

Supplement: Supplementary file 1 [file molecules-18-07346-s001.pdf]
